# Visceral adiposity measures are strongly associated with cardiovascular disease among female participants in Southwest China: A population-based prospective study

**DOI:** 10.3389/fendo.2022.969753

**Published:** 2022-09-08

**Authors:** Yingying Wang, Xiaodeng Zhao, Yun Chen, Yuntong Yao, Yixia Zhang, Na Wang, Tao Liu, Chaowei Fu

**Affiliations:** ^1^ Key Laboratory of Public Health Safety, NHC Key Laboratory of Health Technology Assessment, School of Public Health, Fudan University, Shanghai, China; ^2^ Guizhou Province Center for Disease Prevention and Control, Chronic Disease Prevention and Cure Research Institute, Guiyang, China

**Keywords:** visceral adiposity, anthropometric, cardiovascular disease, southwest China, cohort study

## Abstract

**Background and aims:**

Controversy remains regarding the prediction effects of different adiposity measure indicators for the risk of cardiovascular disease (CVD). Our study aimed to assess the associations of three traditional anthropometric indicators, namely, waist circumference (WC), waist-to-height ratio (WHtR), and body mass index (BMI) as well as three non-traditional anthropometric indicators, namely, the Chinese visceral adiposity index (CVAI), lipid accumulation product (LAP), and body shape index (ABSI), with the risk of CVD among Southwest Chinese population.

**Methods:**

Our study was based on the Guizhou Population Health Cohort Study (GPHCS) conducted from 2010 to 2020. A total of 9,280 participants were recruited from 12 areas in Guizhou Province, China, from November 2010 to December 2012, and followed up for major chronic diseases until December 2020. A total of 7,837 individuals with valid data were included in this analysis. The gender-specific associations of WC, WHtR, BMI, CVAI, LAP, and ABSI with CVD were evaluated using Cox proportional hazards models. Receiver operating characteristic (ROC) curve analysis was used to estimate the prediction powers of different indicators for CVD.

**Results:**

No association of six indicators with CVD was observed among male participants. Female participants with either WC-based central obesity (HR: 1.82, 95% CI: 1.12–2.97) or WHtR-based central obesity (HR: 1.68, 95% CI: 1.07–2.64) had a higher risk of CVD, after adjusted for age, area, ethnic group, smoking, alcohol drinking, MET, previous history of diabetes, hypertension and dyslipidemia, medication use, and nutraceutical intake. Compared with female participants in the lowest quartile (Q1), those in the highest quartile (Q4) of WHtR (HR: 2.24, 95% CI: 1.17–4.27), CVAI (HR: 3.98, 95% CI: 1.87–8.49), and ABSI (HR: 1.94, 95% CI: 1.06–3.52) had an increased risk for incident CVD. CAVI showed the maximum predictive power of CVD with the biggest AUC of 0.687 (95% CI: 0.654–0.720) compared to other indicators in female participants.

**Conclusions:**

Visceral adiposity measures, especially CVAI, are stronger predictive indicators of CVD among female and not male participants in Southwest China. Different anthropometric indexes need to be combined to comprehensively assess health risks.

## Introduction

Cardiovascular disease (CVD) remains the leading cause of mortality and morbidity worldwide, with ischemic heart disease (IHD) and stroke as the main contributors (1). It was estimated that CVD caused 17.8 million deaths in 2017 worldwide (2), and 4 million deaths in 2016 in China (3). The burden of IHD and stroke in China has rapidly and substantially increased during the past two decades (4). IHD caused more than 1 million deaths per year, and the number of individuals with acute myocardial infarction (AMI) will increase to 23 million by 2030 (5). Unlike in Western countries, the epidemic profile of stroke in China surpasses that of IHD, with annual estimates of 11 million prevalent cases, 2.4 million new cases, and 1.1 million deaths (6). Adiposity is an imbalance between energy intake and metabolism expenditure resulting in abnormal fat accumulation. The prevalence of adiposity has reached nearly 33.3% worldwide, doubling since 1980, and is generally highest in developed countries and increasing in Asian countries (7). Two types of obesity, central (visceral) and general (peripheral) obesity, are often assessed by waist circumference (WC) and body mass index (BMI), respectively. Based on BMI criteria, the prevalence was 34.3% for overweight and 16.4% for obesity in Chinese adults (≥18 years) (8).

In fact, the distribution rather than the mount of adipose tissue may have a more critical effect on the development of CVD. Imaging-based assessments of subcutaneous adiposity tissue (SAT) and visceral adiposity tissue (VAT) by routine clinical practices, including computed tomography (CT), magnetic resonance imaging (MRI), dual-energy x-ray absorptiometry (DEXA), and dual bioelectrical impedance analysis (BIA), were largely limited due to their higher costs, related technical challenges, and potential radiation exposure risk. Previous research debated the prediction values of several common anthropometric indicators of adiposity for CVD (9–11); some suggested that waist-to-height ratio (WHtR) was superior to WC and BMI (12, 13). These results had significant heterogeneities as they covered different ethnic populations (14). Recently, visceral adiposity index (VAI), lipid accumulation product (LAP), and body shape index (ABSI), which are the products of WC, BMI, and blood lipids, have been proposed as reliable indexes of body fat accumulation, and they have been applied to the prediction of diabetes (15–17). With reference to VAI and considering the characteristic of body fat in the Asian population, the Chinese visceral adiposity index (CVAI) has been designed for the Chinese population; this surrogate indicator may be more sensitive than VAI, WC, and BMI to discriminate diabetes (18). However, studies investigating the associations between these novel indicators and CVD are limited.

Growing researches suggested that adiposity was significantly associated with CVD and CVD-related risk factors in East China (19–21). The disease burden and risk profiles for CVD vary geographically in China, with higher incidences but less healthcare services in southwestern provinces compared with eastern regions (22–24). However, limited knowledge is available on the effects of adiposity on CVD risk in Southwest China. Guizhou Province lies to the east of the Yunnan-Guizhou Plateau in Southwest China, with complex topography, poor transportation system, and undeveloped economic and educational level, leading to deficiency in medical resources. There are 56 ethnic groups in Guizhou Province, the majority of which are Han. The diet and living habits of different ethnic groups are different, and some prefer pickled food, which increases the risk of hypertension and stroke (25). In this study, we aimed to provide an insight to explore the associations of several anthropometric indicators with cardiovascular onsets, by using data from a large cohort study in Guizhou Province, Southwest China.

## Methods

### Study population and data collection

The Guizhou Population Health Cohort Study (GPHCS) is a good representation of the geographic, socio-demographic, ethnic composition of the adult population in Guizhou Province in Southwest China, enrolling a total of 9,280 adults at baseline between November 2010 and December 2012, from 12 areas (5 urban districts and 7 rural counties) in Guizhou Province using the multistage proportional stratified cluster sampling method, considering population size, population stability, and local capacity. The inclusive criteria were as follows: (1) age of 18 years or above; (2) living locally for more than 6 months and having no plan to move out; (3) completing survey questionnaire, blood sampling, and physical examination; and (4) signing the written informed consent. All participants were followed up for major chronic diseases and vital status through a repeated investigation by trained investigators between 2016 and 2020, and record linkage to the Death Registration Information System and Basic Public Health Service System. Ethics approval was obtained from the ethics review board of Guizhou Province (No.S2017-02).

**Figure 1:**
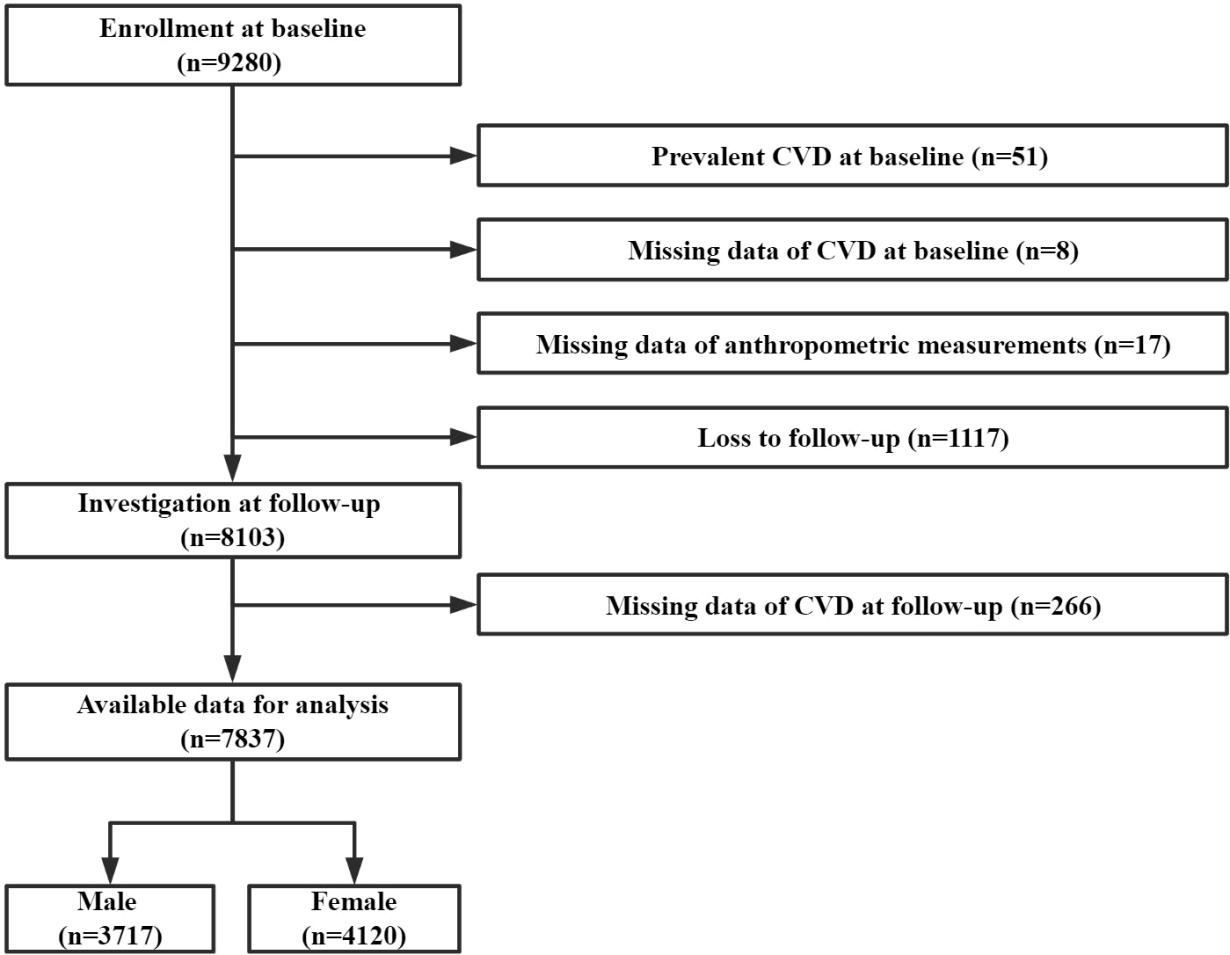
Flowchart of the study.

In this study, we excluded participants with a previous diagnosis of CVD, those with missing data of anthropometric measurements and CVD, or those lost to follow-up. Finally, 7,837 participants were included in the primary analyses (**Figure 1**).

### Outcomes of interest

The primary outcomes were the first occurrence of cardiovascular events, including stroke and IHD. The main types were ischemic stroke (I63), hemorrhagic stroke (I60–61), and myocardial infarction (I21), coded by International Classification of Diseases 10th revision (ICD-10). All reported CVD events were identified using imagological diagnosis by trained clinical staff. The person-year (PY) of follow-up was calculated from the date of baseline investigation to the date of the occurrence of CVD, death, or follow-up, whichever came first. Incidence rate was calculated as the number of incident cases divided by follow-up PYs.

### Anthropometric measurements and laboratory biochemical information

Anthropometric measurements, including standing height (cm), weight (kg), and WC (cm), were taken by trained health professionals according to standard protocols. Standing height and weight were measured with participants standing without shoes and in lightweight clothes. WC was measured on the midaxillary line between the lowest border of the rib cage and the top of the iliac crest. All parameters were recorded as the mean value of the twice measurements, and usually to the nearest 0.1 cm or 0.1 kg. Blood pressure was measured three times in a 3-min interval from the left arm after the participant rests in a seated position; the recorded values of systolic blood pressure (SBP) and diastolic blood pressure (DBP) were calculated as the mean of the last two of three consecutive measurements.

All participants provided a 10-ml blood sample after an overnight fast of at least 10 h, they also undergo an oral glucose tolerance test (OGTT), and the plasma was obtained at 2 h during the test. Concentrations of fasting plasma glucose (FPG), 2-h postload glucose (2h-PG), and Hemoglobin A1c (HbA1c) were analyzed locally within 2 h after the blood sample collected using the glucose oxidase methods (Roche Diagnostics, Mannheim, Germany). Serum triglycerides (TG), total cholesterol (CHOL), low-density lipoprotein cholesterol (LDL-C), and high-density lipoprotein cholesterol (HDL-C) were measured using enzymatic methods (Roche Diagnostics, Mannheim, Germany).

Traditional and non-traditional anthropometric indicators were calculated by the following formula:


(1) WHtR=WC÷height ;

(2) $$\text{BMI}\left(\text{kg}/{\text{m}}^{2}\right)=\text{weight}÷{\left(\text{height}÷100\right)}^{2};$$

(3) CVAI=−267.93+0.68×Age+0.03×BMI+4.00×WC+22.00×LgTG−16.32×HDL(for male participants); CVAI=−187.32+1.71×Age+4.23×BMI+1.12×WC+39.76×Lg TG−11.66×HDL(for female participants);

(4) LAP=(WC−65)×TG(for male participants); LAP=(WC−58)×TG(for female participants);

(5) $$\text{ABSI}=\text{WC}/\left({\text{BMI}}^{2/3}\times {\text{height}}^{1/2}\right).$$

### Other data collections

Standardized in-person interviews using structured questionnaires were conducted for each participant to obtain the socio-demographic (age, gender, area, ethnic group, education level, marriage status, and occupation type), lifestyle (physical activity, tobacco smoking, and alcohol use), comorbidity status (diabetes, hypertension, and dyslipidemia), medication use, and nutraceutical consumption information.

Smoking was defined as smoking at least one cigarette a day for 12 months or more. Alcohol drinking was defined as drinking at least three times a week for 12 months or more. The physical activity level was calculated as the product of the duration and frequency of each activity, weighted by an estimate of the metabolic equivalent (MET) of that activity and summed for all activities performed, with the result expressed as the average MET hours per day. Diabetes was defined as those above the threshold of glycemia (FPG ≥ 6.1 mmol/L or 2h-PG ≥ 7.8 mmol/L), having a reported diabetes history, or experiencing anti-diabetes medications (26). Hypertension was defined as abnormal level of current blood pressure (SBP > 140 mmHg or DBP > 90 mmHg), having a reported hypertension history, or experiencing anti-hypertension medications (26). Dyslipidemia was defined as abnormal level of current blood lipids (TG ≥ 1.7 mmol/L, CHOL ≥ 5.2 mmol/L, LDL ≥ 3.4 mmol/L, HDL < 1.0 mmol/L), having a reported dyslipidemia history, or experiencing anti-dyslipidemia medications (26). Medication use was defined as regularly taking any medications for diabetes (including metformin, insulin, etc.), hypertension (including amlodipine, nifedipine, etc.), dyslipidemia (including atorvastatin, simvastatin, etc.), or obesity. Nutraceutical intake was defined as intaking common nutraceuticals (including vitamin, minerals, etc.) or foods with healthcare functions (including wine, tea, etc.) at least once a week for 12 months or more.

### Statistical analyses

Baseline characteristics for participants were presented according to the presence of incident CVD and compared using Student’s *t*-test for continuous variables, or Chi-square test for categorical variables. Considering the body fat distribution and blood biochemical profiles are distinctly different in male and female participants, all analyses were conducted separately by sex.

The proportional hazard assumption was satisfied and then age-adjusted or multivariate-adjusted Cox proportional hazard models were used to assess the associations of incident CVD with adiposity categories and anthropometric indicators. The corresponding hazard ratios (HRs) and 95% confidence intervals (95% CIs) were calculated. Participants were categorized into different adiposity category groups according to gender-specific cutoffs for WC (normal weight, <85 cm for female participants and <90 cm for male participants; and central obesity, ≥85 cm for female participants and ≥90 cm for male participants) (27), WHtR (normal weight, <0.5; and central obesity, >0.5) (28), and BMI (lower weight, <18.5 kg/m^2^; normal weight, 18.5–23.9 kg/m^2^; overweight, 24.0–27.9 kg/m^2^; and obesity, ≥28.0 kg/m^2^) (27), based on Chinese guidelines. Participants were also categorized into four groups according to the quartiles of traditional (WC, WHtR, and BMI) and non-traditional (CVAI, LAP, and ABSI) anthropometric indicators, respectively. The restricted cubic splines (RCS) in Cox regression analyses were applied to evaluate the potential dose–response relationships of these six anthropometric indicators with CVD events. Receiver operator characteristic (ROC) curve analyses were generated for multivariate-adjusted Cox proportional hazard models, and the predictive powers of six indicators for CVD were compared according to the area under the ROC curve (AUC). Similar analyses were conducted in subgroups stratified by baseline demographic (age, area, and ethnic group).

All analyses and figures were performed by using R program (version 4.1.0, R Foundation for Statistical Computing, Vienna, Austria).

## Results

### Baseline characteristic descriptions

Of the 7,837 participants included, the mean (SD) age was 44.18 ± 14.97 years, and more than half were female (52.57%), ethnically Han (58.52%), and rural residents (67.02%). During the median 6.59 years of follow-up, 193 cases of first-onset CVD were identified (incident rate: 3.47 per 1,000 person-years), including 141 ischemic strokes (incident rate: 2.53 per 1,000 person-years), 46 hemorrhagic strokes (incident rate: 0.82 per 1,000 person-years), and 27 myocardial infarctions (incident rate: 0.48 per 1,000 person-years). Compared with female participants without CVD, those with CVD seemed to experience a higher level of WC, WHtR, CVAI, LAP, and ABSI at baseline (*p* < 0.05). However, there was no significant difference in these indicators between male participants with CVD and those without (**Table 1**).

**Table 1:** Baseline characteristics for participants.

Characteristics	Male participants				Female participants			
	Total (*n* = 3,717)	Non-CVD (*n* = 3,621)	CVD (*n* = 96)	*p*-value	Total (*n* = 4120)	Non-CVD (*n* = 4023)	CVD (*n* = 97)	*p*-value
Age (years, mean ± SD)	43.66 ±15.02	43.41 ± 14.99	53.18 ± 13.34	<0.001	44.64 ± 14.90	44.41 ± 14.85	54.50 ± 13.78	<0.001
Area(*n*,%)				0.305				0.388
Urban	1,206 (32.4)	1,180 (32.6)	26 (27.1)		1,379 (33.5)	1,351 (33.6)	28 (28.9)	
Rural	2,511 (67.6)	2,441 (67.4)	70 (72.9)		2,741 (66.5)	2,672 (66.4)	69 (71.1)	
Ethnic group(*n*,%)				0.060				0.092
Ethnic Han	2,190 (58.9)	2,124 (58.7)	66 (68.8)		2,396 (58.2)	2,331 (57.9)	65 (67.0)	
Minority	1,527 (41.1)	1,497 (41.3)	30 (31.2)		1,724 (41.8)	1,692 (42.1)	32 (33.0)	
Education (*n*,%)				0.455				0.016
No formal education	365 (9.8)	352 (9.7)	13 (13.5)		1,238 (30.0)	1,196 (29.7)	42 (43.3)	
Junior middle school and below	2,772 (74.6)	2,704 (74.7)	68 (70.8)		2,421 (58.8)	2,375 (59.0)	46 (47.4)	
Senior high school and above	580 (15.6)	565 (15.6)	15 (15.6)		461 (11.2)	452 (11.2)	9 (9.3)	
Marriage (*n*,%)				0.226				0.327
Married/Cohabit	2,932 (78.9)	2,851 (78.7)	81 (84.4)		3,403 (82.6)	3,327 (82.7)	76 (78.4)	
Unmarried/Divorced/Widowed/Separated	785 (21.1)	770 (21.3)	15 (15.6)		717 (17.4)	696 (17.3)	21 (21.6)	
Occupation (*n*,%)				0.915				0.045
Farmers	2,182 (58.7)	2,127 (58.7)	55 (57.3)		2,306 (56.0)	2,247 (55.9)	59 (60.8)	
Others	1,118 (30.1)	1,089 (30.1)	29 (30.2)		973 (23.6)	960 (23.9)	13 (13.4)	
Unemployed/Retired	417 (11.2)	405 (11.2)	12 (12.5)		841 (20.4)	816 (20.3)	25 (25.8)	
Smoking (*n*, %)				1.000				0.018
No	1,779 (47.9)	1,733 (47.9)	46 (47.9)		4,074 (98.9)	3,981 (99.0)	93 (95.9)	
Yes	1,938 (52.1)	1,888 (52.1)	50 (52.1)		46 (1.1)	42 (1.0)	4 (4.1)	
Alcohol drinking (*n*, %)				0.786				0.447
No	2,371 (63.8)	2,308 (63.7)	63 (65.6)		3,660 (88.8)	3,571 (88.8)	89 (91.8)	
Yes	1,346 (36.2)	1,313 (36.3)	33 (34.4)		460 (11.2)	452 (11.2)	8 (8.2)	
Diabetes (*n*, %)				0.015				0.182
No	3,359 (90.7)	3,281 (90.9)	78 (83.0)		3,799 (92.6)	3,714 (92.7)	85 (88.5)	
Yes	344 (9.3)	328 (9.1)	16 (17.0)		304 (7.4)	293 (7.3)	11 (11.5)	
Hypertension (*n*, %)				0.001				<0.001
No	2,668 (71.8)	2,614 (72.2)	54 (56.2)		3,162 (76.7)	3,108 (77.3)	54 (55.7)	
Yes	1,049 (28.2)	1,007 (27.8)	42 (43.8)		958 (23.3)	915 (22.7)	43 (44.3)	
Dyslipidemia (*n*, %)				0.322				0.980
No	1,636 (44.0)	1,599 (44.2)	37 (38.5)		1,715 (41.6)	1,674 (41.6)	41 (42.3)	
Yes	2,081 (56.0)	2,022 (55.8)	59 (61.5)		2,405 (58.4)	2,349 (58.4)	56 (57.7)	
Medication use (*n*, %) ^a^				0.943				0.760
No	3,263 (87.8)	3,178 (87.8)	85 (88.5)		3,589 (87.1)	3,506 (87.1)	83 (85.6)	
Yes	454 (12.2)	443 (12.2)	11 (11.5)		531 (12.9)	517 (12.9)	14 (14.4)	
Nutraceutical intake (*n*, %) ^a^				0.858				1.000
No	3,282 (88.5)	3,196 (88.5)	86 (89.6)		3,647 (88.7)	3,561 (88.7)	86 (88.7)	
Yes	427 (11.5)	417 (11.5)	10 (10.4)		465 (11.3)	454 (11.3)	11 (11.3)	
WC-based				1				<0.001
Normal weight	3,068 (87.3)	2,992 (87.3)	76 (87.4)		3,211 (83.8)	3,149 (84.1)	62 (69.7)	
Central obesity	446 (12.7)	435 (12.7)	11 (12.6)		623 (16.2)	596 (15.9)	27 (30.3)	
WHtR-based				0.870				0.001
Normal weight	2,334 (66.4)	2,275 (66.4)	59 (67.8)		2,168 (56.5)	2,133 (57.0)	35 (39.3)	
Central obesity	1,180 (33.6)	1,152 (33.6)	28 (32.2)		1,666 (43.5)	1,612 (43.0)	54 (60.7)	
BMI-based				0.671				0.064
Underweight	189 (5.1)	182 (5.0)	7 (7.3)		238 (5.8)	236 (5.9)	2 (2.1)	
Normal weight	2,386 (64.2)	2,329 (64.3)	57 (59.4)		2,501 (60.7)	2,438 (60.6)	63 (64.9)	
Overweight	915 (24.6)	889 (24.6)	26 (27.1)		1,044 (25.3)	1,025 (25.5)	19 (19.6)	
Obesity	227 (6.1)	221 (6.1)	6 (6.2)		337 (8.2)	324 (8.1)	13 (13.4)	
WC (cm, mean ± SD) ^a^	77.92 ± 9.49	77.92 ± 9.48	77.74 ± 9.80	0.858	75.42 ± 9.30	75.35 ± 9.27	78.37 ± 9.91	0.002
WHtR (mean ± SD) ^a^	0.48 ± 0.06	0.48 ± 0.06	0.48 ± 0.06	0.820	0.49 ± 0.06	0.49 ± 0.06	0.52 ± 0.07	<0.001
BMI (kg/m^2^, mean ± SD)	22.77 ± 3.15	22.77 ± 3.15	22.81 ± 3.10	0.911	22.99 ± 3.40	22.98 ± 3.40	23.55 ± 3.32	0.105
CVAI (mean ± SD)	53.52 ± 44.06	53.37 ± 44.03	59.39 ± 44.92	0.211	61.47 ± 42.57	60.86 ± 42.37	87.25 ± 43.32	<0.001
LAP (mean ± SD)	28.49 ± 47.34	28.53 ± 47.48	26.66 ± 41.83	0.717	32.36 ± 39.63	32.07 ± 38.66	44.93 ± 68.26	0.003
ABSI (m^11/6^/kg^2/3,^ mean ± SD)	0.76 ± 0.06	0.76 ± 0.06	0.76 ± 0.05	0.801	0.76 ± 0.06	0.76 ± 0.06	0.78 ± 0.06	0.006
FPG (mmol/L, mean ± SD) ^a^	5.32 ± 1.38	5.32 ± 1.37	5.46 ± 1.70	0.331	5.19 ± 1.13	5.19 ± 1.13	5.33 ± 1.41	0.205
2h-PG (mmol/L, mean ± SD) ^a^	5.87 ± 2.41	5.86 ± 2.40	6.42 ± 2.78	0.026	5.72 ± 2.10	5.71 ± 2.09	5.91 ± 2.20	0.350
SBP (mmHg, mean ± SD) ^a^	126.99 ± 20.36	126.78 ± 20.24	134.93 ± 23.24	<0.001	123.37 ± 21.18	123.11 ± 20.93	134.32 ± 27.45	<0.001
DBP (mmHg, mean ± SD) ^a^	79.30 ± 11.83	79.19 ± 11.76	83.50 ± 13.85	<0.001	77.28 ± 11.88	77.17 ± 11.80	82.02 ± 14.33	<0.001
TG (mmol/L, mean ± SD) ^a^	1.84 ± 1.81	1.84 ± 1.80	1.95 ± 2.04	0.552	1.68 ± 1.31	1.67 ± 1.28	1.94 ± 2.02	0.050
CHOL (mmol/L, mean ± SD) ^a^	4.78 ± 1.26	4.77 ± 1.24	4.96 ± 1.83	0.157	4.81 ± 1.37	4.80 ± 1.36	5.01 ± 1.65	0.134
HDL-C (mmol/L, mean ± SD)	1.45 ± 0.54	1.45 ± 0.54	1.44 ± 0.55	0.817	1.45 ± 0.57	1.45 ± 0.56	1.41 ± 0.65	0.452
LDL-C (mmol/L, mean ± SD) ^a^	2.64 ± 1.13	2.64 ± 1.13	2.51 ± 1.22	0.271	2.68 ± 1.23	2.68 ± 1.23	2.69 ± 1.48	0.921
MET (per day, *n*, %) ^a^	117.18 ± 127.77	117.05 ± 127.92	121.79 ± 122.73	0.720	103.11 ± 117.32	103.13 ± 117.37	102.33 ± 115.77	0.947

CVD, cardiovascular diseases; SD, standard deviation; WC, waist circumference; WHtR, waist-to-height ratio; BMI, body mass index; CVAI, Chinese visceral adiposity index; LAP, lipid accumulation product; ABSI, body shape index; FPG, fasting plasma glucose; 2h-PG, 2-h postload glucose; SBP, systolic blood pressure; DBP, diastolic blood pressure; TG, triglyceride; CHOL, total cholesterol; HDL-C, high-density lipoprotein cholesterol; LDL-C, low-density lipoprotein cholesterol; MET, metabolic equivalent of task.

a With missing value.

### Major analyses

Cox proportional hazard models indicated no association between any obesity type and incident CVD among male participants (**Tables 2**, **3**). Female participants with either WC-based central obesity (HR:1.83, 95% CI: 1.12–2.98) or WHtR-based central obesity (HR:1.69, 95% CI: 1.07–2.65) had a higher risk of CVD after adjusting for age, area, ethnic group, smoking, alcohol drinking, MET, previous history of diabetes, hypertension, and dyslipidemia (**Table 2**, Model 2). The effect sizes of these positive associations were slightly decreased when further adjusted for medication use and nutraceutical intake (WC-based, HR: 1.82, 95% CI: 1.12–2.97; WHtR-based, HR:1.68, 95% CI: 1.07–2.64; **Table 2**, Model 3).

**Table 2:** Hazard ratios (HRs) and 95% confidence intervals (95% CIs) for overall CVD associated with adiposity category among male (*n* = 3,717) and female (*n* = 4,120) participants according to Cox regression models.

Anthropometric indexes	No (*n*)	Cases (*n*)	Incident density (cases per 1,000 PYs)	HR (95% CI) ^a^		
				Model 1	Model 2	Model 3
**Male participants**						
**WC-based**						
Normal weight	3,068	76	3.45	1.00	1.00	1.00
Central obesity	446	11	3.50	1.04 (0.55–1.96)	0.84 (0.44–1.63)	0.85 (0.44–1.63)
**WHtR-based**						
Normal weight	2,334	59	3.52	1.00	1.00	1.00
Central obesity	1,180	28	3.34	0.97 (0.62–1.52)	0.82 (0.51–1.32)	0.82 (0.51–1.32)
**BMI-based**						
Underweight	189	7	5.24	1.59 (0.73–3.49)	1.57 (0.67–3.66)	1.58 (0.68–3.68)
Normal weight	2,386	57	3.36	1.00	1.00	1.00
Overweight	915	26	3.99	1.18 (0.74–1.87)	1.04 (0.65–1.68)	1.04 (0.65–1.68)
Obesity	227	6	3.81	1.15 (0.50–2.67)	0.84 (0.35–2.01)	0.84 (0.35–2.01)
**Female participants**						
**WC-based**						
Normal weight	3,211	62	1.37	1.00	1.00	1.00
Central obesity	623	27	3.59	2.35 (1.50–3.70)***	1.83 (1.12–2.98)*	1.82 (1.12–2.97)*
**WHtR-based**						
Normal weight	2,168	35	1.08	1.00	1.00	1.00
Central obesity	1,666	54	2.68	2.09 (1.37–3.20)***	1.69 (1.07–2.65)*	1.68 (1.07–2.64)*
**BMI-based**						
Underweight	238	2	0.65	0.31 (0.08–1.28)	0.32 (0.08–1.31)	0.33 (0.08–1.33)
Normal weight	2,501	63	1.81	1.00	1.00	1.00
Overweight	1,044	19	1.37	0.73 (0.43–1.21)	0.56 (0.33–0.96)	0.56 (0.32–0.96)
Obesity	337	13	3.32	1.58 (0.87–2.87)	1.05 (0.56–1.99)	1.06 (0.56–2.00)

CVD, cardiovascular and cerebrovascular diseases; WC, waist circumference; WHtR, waist-to-height ratio; BMI, body mass index; PYs, person-years; MET, metabolic equivalent of task.

a **Model 1:** Adjusted for age only; **Model 2:** Model 1 **+** additionally adjusted for area, ethnic group, smoking, alcohol drinking, MET, diabetes, hypertension, and dyslipidemia,; **Model 3**: Model 2 **+** additionally adjusted for medication use and nutraceutical intake.

*0.01 < p < 0.05; ***p <0.001.

**Table 3:** Hazard ratios (HRs) and 95% confidence intervals (95% CIs) for overall CVDs associated with traditional and non-traditional anthropometric indicators among male participants (*n* = 3,717) according to Cox regression models.

Anthropometric indicators	No (*n*)	Cases (*n*)	Incident density (cases per 1,000 PYs)	HR (95% CI) ^a^		
				Model 1	Model 2	Model 3
**Traditional**						
**WC (cm) ^b^**						
Quartile 1 (Q1)	843	23	3.80	1.00	1.00	1.00
Quartile 2 (Q2)	914	17	2.59	0.68 (0.36–1.27)	0.62 (0.32–1.17)	0.61 (0.32–1.17)
Quartile 3 (Q3)	857	23	3.73	1.00 (0.56–1.78)	0.92 (0.51–1.67)	0.92 (0.51–1.68)
Quartile 4 (Q4)	900	24	3.77	1.00 (0.57–1.78)	0.80 (0.43–1.48)	0.80 (0.43–1.48)
*p* trend	–	–	–	0.687	0.783	0.788
Per 1 SD	–	–	–	1.00 (0.81–1.23)	0.91 (0.73–1.14)	0.91 (0.73–1.14)
**WHtR ^c^**						
Quartile 1 (Q1)	878	23	3.64	1.00	1.00	1.00
Quartile 2 (Q2)	879	21	3.31	0.93 (0.51–1.67)	0.86 (0.47–1.57)	0.85 (0.46–1.57)
Quartile 3 (Q3)	878	18	2.87	0.82 (0.44–1.52)	0.74 (0.40–1.40)	0.74 (0.40–1.40)
Quartile 4 (Q4)	879	25	4.02	1.15 (0.65–2.02)	0.92 (0.50–1.69)	0.92 (0.50–1.70)
*p* trend	–	–	–	0.733	0.720	0.730
Per 1 SD	–	–	–	1.00 (0.81–1.24)	0.92 (0.73–1.15)	0.92 (0.73–1.15)
**BMI (kg/m^2^) ^d^**						
Quartile 1 (Q1)	929	24	3.62	1.00	1.00	1.00
Quartile 2 (Q2)	929	21	3.17	0.87 (0.48–1.55)	0.81 (0.45–1.49)	0.81 (0.45–1.48)
Quartile 3 (Q3)	925	27	4.12	1.12 (0.65–1.94)	1.03 (0.58–1.80)	1.02 (0.58–1.80)
Quartile 4 (Q4)	934	24	3.64	1.00 (0.57–1.76)	0.81 (0.44–1.48)	0.81 (0.44–1.48)
*p* trend	–	–	–	0.776	0.675	0.673
Per 1 SD	–	–	–	1.02 (0.83–1.24)	0.93 (0.75–1.16)	0.93 (0.75–1.16)
**Non-traditional**						
**CAVI ^e^**						
Quartile 1 (Q1)	865	17	2.74	1.00	1.00	1.00
Quartile 2 (Q2)	865	18	2.89	1.04 (0.54–2.02)	1.00 (0.51–2.00)	1.00 (0.51–1.99)
Quartile 3 (Q3)	865	23	3.70	1.36 (0.73–2.55)	1.27 (0.66–2.46)	1.27 (0.66–2.45)
Quartile 4 (Q4)	866	28	4.59	1.68 (0.92–3.06)	1.41 (0.72–2.77)	1.42 (0.72–2.78)
*p* trend	–	–	–	0.056	0.239	0.235
Per 1 SD	–	–	–	1.15 (0.94–1.41)	1.04 (0.83–1.31)	1.04 (0.83–1.31)
**LAP ^f^**						
Quartile 1 (Q1)	870	20	3.20	1.00	1.00	1.00
Quartile 2 (Q2)	868	18	2.91	0.91 (0.48–1.71)	0.88 (0.46–1.69)	0.88 (0.46–1.68)
Quartile 3 (Q3)	869	30	4.83	1.54 (0.88–2.71)	1.34 (0.74–2.43)	1.33 (0.74–2.41)
Quartile 4 (Q4)	872	18	2.88	0.90 (0.48–1.70)	0.70 (0.35–1.41)	0.70 (0.35–1.40)
*p* trend	–	–	–	0.762	0.639	0.630
Per 1 SD	–	–	–	0.97 (0.76–1.22)	0.87 (0.66–1.15)	0.88 (0.66–1.16)
**ABSI ^g^**						
Quartile 1 (Q1)	879	21	3.33	1.00	1.00	1.00
Quartile 2 (Q2)	878	25	4.00	1.23 (0.69–2.20)	1.31 (0.72–2.38)	1.31 (0.72–2.38)
Quartile 3 (Q3)	878	22	3.47	1.08 (0.59–1.97)	1.09 (0.59–2.03)	1.10 (0.59–2.04)
Quartile 4 (Q4)	879	19	3.04	0.97 (0.52–1.81)	0.90 (0.47–1.71)	0.90 (0.47–1.72)
*p* trend	–	–	–	0.835	0.605	0.614
Per 1 SD	–	–	–	1.00 (0.81–1.24)	0.97 (0.77–1.20)	0.97 (0.78–1.20)

CVD, cardiovascular and cerebrovascular diseases; WC, waist circumference; WHtR, waist-to-height ratio; BMI, body mass index; CVAI, Chinese visceral adiposity index; LAP, lipid accumulation product; ABSI, body shape index; PYs, person-years; MET, metabolic equivalent of task.

a **Model 1:** Adjusted for age only; **Model 2:** Model 1 **+** additionally adjusted for area, ethnic group, smoking, alcohol drinking, MET, diabetes, hypertension and dyslipidemia; **Model 3**: Model 2 **+** additionally adjusted for medication use and nutraceutical intake.

b **WC (cm):** Quartile levels as Q1, <71.00 cm; Q2, 71.00–76.54 cm; Q3, 76.55–84.39 cm; Q4, ≥84.00 cm

c **WHtR:** Quartile levels as Q1, <0.44; Q2, 0.44–0.47; Q3, 0.48–0.51; Q4, ≥0.52.

d **BMI (kg/m^2^):** Quartile levels as Q1, <20.47 kg/m^2^; Q2, 20.47–22.34 kg/m^2^; Q3, 22.35–24.56 kg/m^2^; Q4, ≥24.57 kg/m^2^.

e **CAVI:** Quartile levels as Q1, <21.30; Q2, 21.30–47.73; Q3, 47.73–82.16; Q4, ≥82.15.

f **LAP:** Quartile levels as Q1, <6.29; Q2, 6.29–14.60; Q3, 14.60–33.60; Q4, ≥33.60.

g **ABSI:** Quartile levels as Q1, <0.73; Q2, 0.73– 0.76; Q3, 0.76–0.80; Q4, ≥0.80.

The dose–response relationships of CVD with WC, WHtR, BMI, CVAI, LAP, and ABSI appeared to follow non-linear patterns among two gender groups (**Figures S1, S2**). Details regarding the associations of CVD risks and six anthropometric indicators are provided in **Tables 3**, **4**. Similarly, these associations were seen only among female and not male participants. Compared with female participants in the lowest quartile (Q1), those in the highest quartile (Q4) of WHtR (HR: 2.24, 95% CI: 1.17–4.27), CVAI (HR: 3.98, 95% CI: 1.87–8.49), and ABSI (HR: 1.94, 95% CI: 1.06–3.52) had an increased risk of incident CVD (**Table 4**, Model 3). Additionally, per 1 SD increase in WHtR, CVAI, LAP, and ABSI increased 32%, 74%, 19%, and 26% risk of CVD, respectively (**Table 4**, Model 3). However, regardless of being evaluated in any form, BMI was unrelated to incident CVD.

**Table 4:** Hazard ratios (HRs) and 95% confidence intervals (95% CIs) for overall CVDs associated with traditional and non-traditional anthropometric indicators among female participants (*n* = 4,120) according to Cox regression models.

Anthropometric indicators	No (*n*)	Cases (*n*)	Incident density (cases per 1,000 PYs)	HR (95% CI) ^a^		
				Model 1	Model 2	Model 3
**Traditional**						
**WC (cm) ^b^**						
Quartile 1 (Q1)	935	15	2.20	1.00	1.00	1.00
Quartile 2 (Q2)	974	19	2.70	1.24 (0.63–2.43)	1.18 (0.60–2.32)	1.18 (0.60–2.32)
Quartile 3 (Q3)	962	20	2.91	1.34 (0.69–2.63)	1.14 (0.58–2.25)	1.14 (0.58–2.25)
Quartile 4 (Q4)	963	35	5.16	2.39 (1.30–4.37)**	1.74 (0.92–3.30)	1.74 (0.92–3.29)
*p* trend	–	–	–	0.003	0.088	0.090
Per 1 SD	–	–	–	1.37 (1.13–1.66)**	1.22 (0.99–1.50)	1.22 (0.99–1.50)
**WHtR ^c^**						
Quartile 1 (Q1)	959	14	1.99	1.00	1.00	1.00
Quartile 2 (Q2)	957	19	2.75	1.41 (0.71–2.81)	1.35 (0.68–2.70)	1.36 (0.68–2.71)
Quartile 3 (Q3)	959	17	2.49	1.28 (0.63–2.60)	1.14 (0.56–2.33)	1.14 (0.56–2.32)
Quartile 4 (Q4)	959	39	5.78	2.98 (1.62–5.48)***	2.23 (1.17–4.26)*	2.24 (1.17–4.27)*
*p* trend	–	–	–	<0.001	0.017	0.017
Per 1 SD	–	–	–	1.47 (1.21–1.78)***	1.32 (1.07–1.63)**	1.32 (1.07–1.63)**
**BMI (kg/m^2^) ^d^**						
Quartile 1 (Q1)	1030	22	2.97	1.00	1.00	1.00
Quartile 2 (Q2)	1030	18	2.44	0.83 (0.44–1.54)	0.82 (0.44–1.53)	0.82 (0.44–1.53)
Quartile 3 (Q3)	1030	27	3.71	1.25 (0.71–2.20)	1.10 (0.62–1.94)	1.10 (0.62–1.95)
Quartile 4 (Q4)	1030	30	4.17	1.41 (0.81–2.44)	1.00 (0.55–1.81)	1.00 (0.55–1.81)
*p* trend	–	–	–	0.107	0.778	0.779
Per 1 SD	–	–	–	1.18 (0.98–1.42)	1.05 (0.85–1.28)	1.05 (0.85–1.28)
**Non-traditional**						
**CAVI ^e^**						
Quartile 1 (Q1)	941	10	1.46	1.00	1.00	1.00
Quartile 2 (Q2)	940	12	1.75	1.19 (0.52–2.76)	1.19 (0.51–2.76)	1.19 (0.51–2.76)
Quartile 3 (Q3)	941	22	3.29	2.25 (1.07–4.75)*	2.27 (1.06–4.87)*	2.26 (1.05–4.84)*
Quartile 4 (Q4)	941	43	6.51	4.44 (2.23–8.84)***	4.01 (1.88–8.54)***	3.98 (1.87–8.49)***
*p* trend	–	–	–	<0.001	<0.001	<0.001
Per 1 SD	–	–	–	1.79 (1.47–2.19)***	1.75 (1.38–2.21)***	1.74 (1.37–2.21)***
**LAP ^f^**						
Quartile 1 (Q1)	945	15	2.20	1.00	1.00	1.00
Quartile 2 (Q2)	947	19	2.81	1.27 (0.65–2.50)	1.23 (0.62–2.42)	1.23 (0.63–2.43)
Quartile 3 (Q3)	945	21	3.10	1.41 (0.73–2.74)	1.24 (0.63–2.44)	1.23 (0.62–2.42)
Quartile 4 (Q4)	952	32	4.69	2.10 (1.14–3.88)*	1.75 (0.89–3.42)	1.74 (0.89–3.40)
*p* trend	–	–	–	0.014	0.111	0.116
Per 1 SD	–	–	–	1.21 (1.08–1.37)**	1.18 (1.03–1.36)*	1.19 (1.03–1.37)*
**ABSI ^g^**						
Quartile 1 (Q1)	959	16	2.31	1.00	1.00	1.00
Quartile 2 (Q2)	958	18	2.60	1.13 (0.58–2.22)	1.11 (0.57–2.19)	1.12 (0.57–2.20)
Quartile 3 (Q3)	958	19	2.78	1.23 (0.63–2.39)	1.14 (0.58–2.22)	1.15 (0.59–2.25)
Quartile 4 (Q4)	959	36	5.30	2.37 (1.32–4.28)**	1.92 (1.06–3.49)*	1.94 (1.06–3.52)*
*p* trend	–	–	–	0.002	0.025	0.023
Per 1 SD	–	–	–	1.35 (1.11–1.63)**	1.26 (1.03–1.54)*	1.26 (1.03–1.54)*

CVD, cardiovascular and cerebrovascular diseases; WC, waist circumference; WHtR, waist-to-height ratio; BMI, body mass index; CVAI, Chinese visceral adiposity index; LAP, lipid accumulation product; ABSI, body shape index; PYs, person-years; MET, metabolic equivalent of task.

a **Model 1:** Adjusted for age only; **Model 2:** Model 1 **+** additionally adjusted for area, ethnic group, smoking, alcohol drinking, MET, diabetes, hypertension and dyslipidemia; **Model 3**: Model 2 **+** additionally adjusted for medication use and nutraceutical intake.

b **WC (cm):** Quartile levels as Q1, <69.00 cm; Q2, 69.00–74.19 cm; Q3, 74.20–80.99 cm; Q4, ≥81.00 cm.

c **WHtR:** Quartile levels as Q1, <0.45; Q2, 0.45–0.48; Q3, 0.49–0.52; Q4, ≥0.53.

d **BMI (kg/m^2^):** Quartile levels as Q1, <20.59 kg/m^2^; Q2, 20.59–22.47 kg/m^2^; Q3, 22.48–24.95 kg/m^2^; Q4, ≥24.96 kg/m^2^.

e **CAVI:** Quartile levels as Q1, <30.46; Q2, 30.46–58.53; Q3, 58.53–91.25; Q4, ≥91.25.

f **LAP:** Quartile levels as Q1, <11.20; Q2, 11.20–20.93; Q3, 20.93–40.00; Q4, ≥40.00.

g **ABSI:** Quartile levels as Q1, <0.72; Q2, 0.72–0.76; Q3, 0.76–0.79; Q4, ≥0.79.

*p < 0.05; **0.05 < p < 0.01; ***0.01 < p < 0.001.

Moreover, the HRs for ischemic stroke, hemorrhagic stroke, and myocardial infarction are presented in **Tables S1–S6**. Compared with female participants in the lowest quartile (Q1), those in the highest quartile (Q4) of CAVI (HR: 3.40, 95% CI: 1.56–7.44) had an elevated risk of ischemic stroke (**Table S2**, Model 3). In addition, CVAI and ABSI were positively associated with the risk of hemorrhagic stroke (**Table S4**, Model 3).

**Figure 2:**
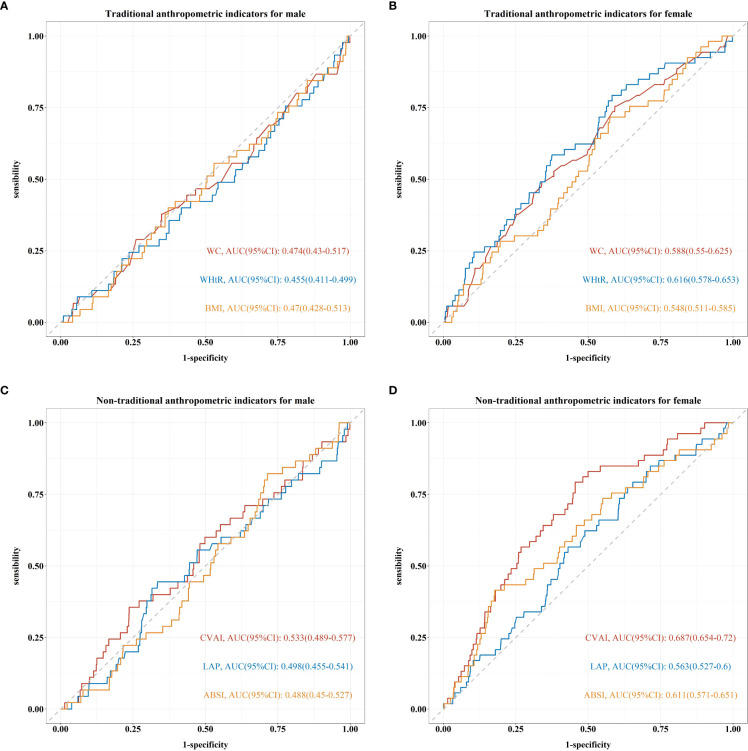
Receiver operating characteristic (ROC) curve and area under ROC curve (AUC) of traditional and non-traditional anthropometric indicators for predicting CVD among male and female participants based on the adjusted Cox regression model (Model 3). **(A, B)** For waist circumference (WC), waist-to-height ratio (WHtR), and body mass index (BMI), respectively. **(C, D)** For Chinese visceral adiposity index (CVAI), lipid accumulation product (LAP), and body shape index (ABSI).

**Figure 2** shows the ROC curves of six indicators in the prediction of CVD among male and female participants, respectively. Neither traditional nor non-traditional indicators predicted CVD in male participants (*p* > 0.05, **Figures 2A, C**). On the contrary, the area under the ROC curve (AUC) and 95% CI for each indicator were higher than 0.5 in female participants (*p* < 0.05, **Figures 2B, D**). CAVI showed the maximum predictive power of CVD with the biggest AUC of 0.687 (95% CI: 0.654–0.720) compared to other indicators in female participants.

### Sensitive analyses and stratified analyses

Sensitive analyses were conducted after excluding female participants with less than 1 year of follow-up, and the results were similar to those in major analyses (**Figure S3**).

**Figure 3:**
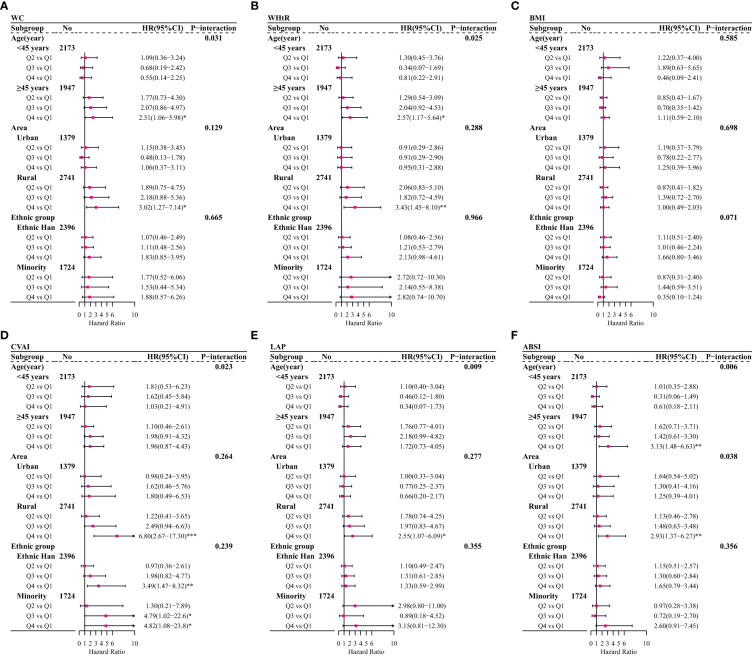
Adjusted hazard ratios (HRs) and 95% confidence intervals (95% CIs) for overall CVD or overall CVD associated with traditional and non-traditional anthropometric indicators among female participants after stratified by age, area, and ethnic group based on adjusted Cox regression model (Model 3). **(A)** For waist circumference (WC); **(B)** for waist-to-height ratio (WHtR); **(C)** for body mass index (BMI); **(D)** for Chinese visceral adiposity index (CVAI); **(E)** for lipid accumulation product (LAP); **(F)** for body shape index (ABSI); *p < 0.05; **0.05 < *p <*0.01;***0.01 < *p* < 0.001.

The multiple-adjusted HRs for incident CVD among female participants predicted by six anthropometric indicators varied according to age, area, and ethnic group (**Figures 3A–F**). The associations of WC and CVD were only observed in female participants aged more than 45 years (HR: 2.51, 95% CI: 1.06–5.98, Q4 vs. Q1) and living in rural region (HR: 3.02, 95% CI: 1.27–7.14, Q4 vs. Q1) (**Figure 3A**). Similar patterns were also seen for WHtR (**Figure 3B**) and ABSI (**Figure 3E**). The most frequent and strongest associations with CVD were found for CVAI, with the HR exceeding 6 (HR: 6.80, 95% CI: 2.67–17.30) in rural residents (**Figure 3D**).

## Discussion

In this large population-based cohort study of 7,837 people with a median of 6.59 years of follow-up in Southwest China, we observed that visceral adiposity measures, especially CVAI, were positively associated with overall CVD and ischemic stroke among female and not male participants. On the contrary, BMI, as a general obesity indicator, performed less predictive power for CVD.

The results of adiposity increasing the risks of CVD in this study are in accordance with those on previous studies (29–31). Adipose tissues release cytokines and chemokines into the vasculature, promoting systemic and vascular inflammation (32). Consistent with our results, obvious sex-related disparities in the associations of adiposity with CVD risk have also been proposed before (29), which may arise not only from differences in body fat distribution and metabolic profiles, but also from the differences in vascular anatomy and physiology, with female participants having smaller arterial diameter than male participants seen after normalizing for body size (33).

WC and WHtR are the most common indicators to measure visceral obesity. WHtR was designed to incorporate the effects of WC and height, namely, WC adjusted for height. There were more frequent associations of CVD with WHtR than WC in this study, suggesting that the distribution of body fat is important in discriminating CVD risk (34). We observed that BMI showed less prediction information for CVD as compared to WC or WHtR. The detrimental vascular effects of adiposity may be masked when using BMI as a measure of adiposity, which has been termed the “obesity paradox”, due to methodological deficiencies such as BMI failing to distinguish between fat tissue and skeletal muscle. In fact, an increase in total fat tissue percent or a decrease in skeletal muscle accelerates the occurrence of CVD (15, 35).

Moreover, the effects of visceral adipose tissue (VAT) on cardio-metabolic outcomes have been proved to be more deleterious than subcutaneous adipose tissue (SAT) (36). CVAI, which is estimated by synergistically integrating information of age, BMI, WC, and lipid profiles (HDL-C and TG), has outperformed traditional anthropometric measures as a useful surrogate for visceral adiposity in a Chinese population (18). As expected, CVAI showed the maximum predictive power of CVD, with a maximum HR of 3.98 (95% CI: 1.87–8.49) and the biggest AUC of 0.687 (95% CI: 0.654–0.720), compared to other indicators in female participants. Previous studies reported that CVAI was superior to BMI, WC, WHtR, LAP, or VAI for the diagnosis of diabetes and related complications (18, 20, 37). Additionally, after combining multiple measurements, ABSI (given the metrics of WC, height, and weight) and LAP (given the metrics of WC and TG) have also been considered as applicable indicators of some chronic diseases in adults (38, 39). In general, several measure indicators need to be combined to comprehensively assess health risks.

In the stratified analyses, the positive associations between visceral adiposity indicators of CVD were stronger in female participants aged more than 45 years or living in rural regions. The mechanism through which adiposity leads to cardiovascular risk is also discrepant in female participants between their pre-menopausal, pregnancy, and post-menopausal phases of life (40). Middle-aged female participants were more likely to accumulate fat due to declines in basal metabolic rate, and the estrogen deprivation secondary to menopause may lead to adverse cardiovascular consequences (41). Area and ethnic variations could be partly explained by regional environmental, socioeconomic characteristics, diet cultures, and local customs (22).

To our knowledge, this is one of few population-based cohort studies to assess CVD risk by a series of adiposity measure indicators. The strengths of our study include covering standardized methods for anthropometric measurements and local residents from various ethnic groups. There were several limitations to this study. First, assessments of some factors in this study, including physical activities, tobacco smoking, alcohol drinking, medications, and nutraceutical consumption, rely on self-reports from questionnaires, which might be influenced by recall bias. Second, we failed to collect any information of medication use for CVD, which have possible beneficial impact on CVD. Third, although the rate of loss to follow-up is above 10%, this rate is relatively low in all studies in Southwest China, given their poor traffic accessibility. Another thing to note is that our study population was from Southeast China, and CVAI was applicable to Chinese people; thus, the findings from this study should be generalized to other populations with caution.

In summary, our study contributes to a new knowledge about the associations of adiposity with incident CVD among Southwest Chinese across a variety of anthropometric indicators. Although visceral adiposity measure indicators are not diagnostic tools for cardiovascular events, the simplicity of anthropometric measurements (WC and BMI) and blood biochemical tests (TG and HDL) might therefore make them well applicable indicators for assessing CVD risk in clinical practice.

## Conclusions

Visceral adiposity measures, especially CVAI, are stronger indicators of CVD among female not male participants in Southwest China. Different anthropometric indicators need to be combined to comprehensively assess health risks.

## Data availability statement

The datasets for this manuscript will be made available upon request, further inquiries can be directed to the corresponding author TL, liutaombs@163.com and NW, na.wang@fudan.edu.cn.

## Ethics statement

The studies involving human participants were reviewed and approved by the ethics review board of Guizhou Province (No. S2017-02). The patients/participants provided their written informed consent to participate in this study.

## Author contributions

Conceptualization: NW, TL, and CF. Data curation: XZ and YC. Investigation: XZ, YY, and YZ. Methodology and formal analysis: YW and NW. Writing—original draft: YW and XZ. Writing—review and editing: NW, TL, and CF. All authors contributed to the preparation of the final document, read, and approved the final manuscript.

## Funding

This work was supported by Guizhou Province Science and Technology Support Program (Qiankehe [2018]2819).

## Conflict of interest

The authors declare that the research was conducted in the absence of any commercial or financial relationships that could be construed as a potential conflict of interest.

## Publisher’s note

All claims expressed in this article are solely those of the authors and do not necessarily represent those of their affiliated organizations, or those of the publisher, the editors and the reviewers. Any product that may be evaluated in this article, or claim that may be made by its manufacturer, is not guaranteed or endorsed by the publisher.

## Abbreviations

CVD, cardiovascular disease; SD, standard deviation; WC, waist circumference; WHtR, waist-to-height ratio; BMI, body mass index; CVAI, Chinese visceral adiposity index; LAP, lipid accumulation product; ABSI, body shape index; FPG, fasting plasma glucose; 2h-PG, 2-h postload glucose; TG, triglyceride; CHOL, total cholesterol; HDL-C, high-density lipoprotein cholesterol; LDL-C, low-density lipoprotein cholesterol; SBP, systolic blood pressure; DBP, diastolic blood pressure; MET, metabolic equivalent of task; ROC, receiver operator characteristic; AUC, area under the ROC curve.
