# Supplementary material for: Visceral adiposity measures are strongly associated with cardiovascular disease among female participants in Southwest China: A population-based prospective study
Source: Front Endocrinol (Lausanne). 2022 Sep 8;13:969753. doi: 10.3389/fendo.2022.969753 (PMC9493204; doi:10.3389/fendo.2022.969753)
Supplement: Supplementary file 1 [file DataSheet_1.pdf]

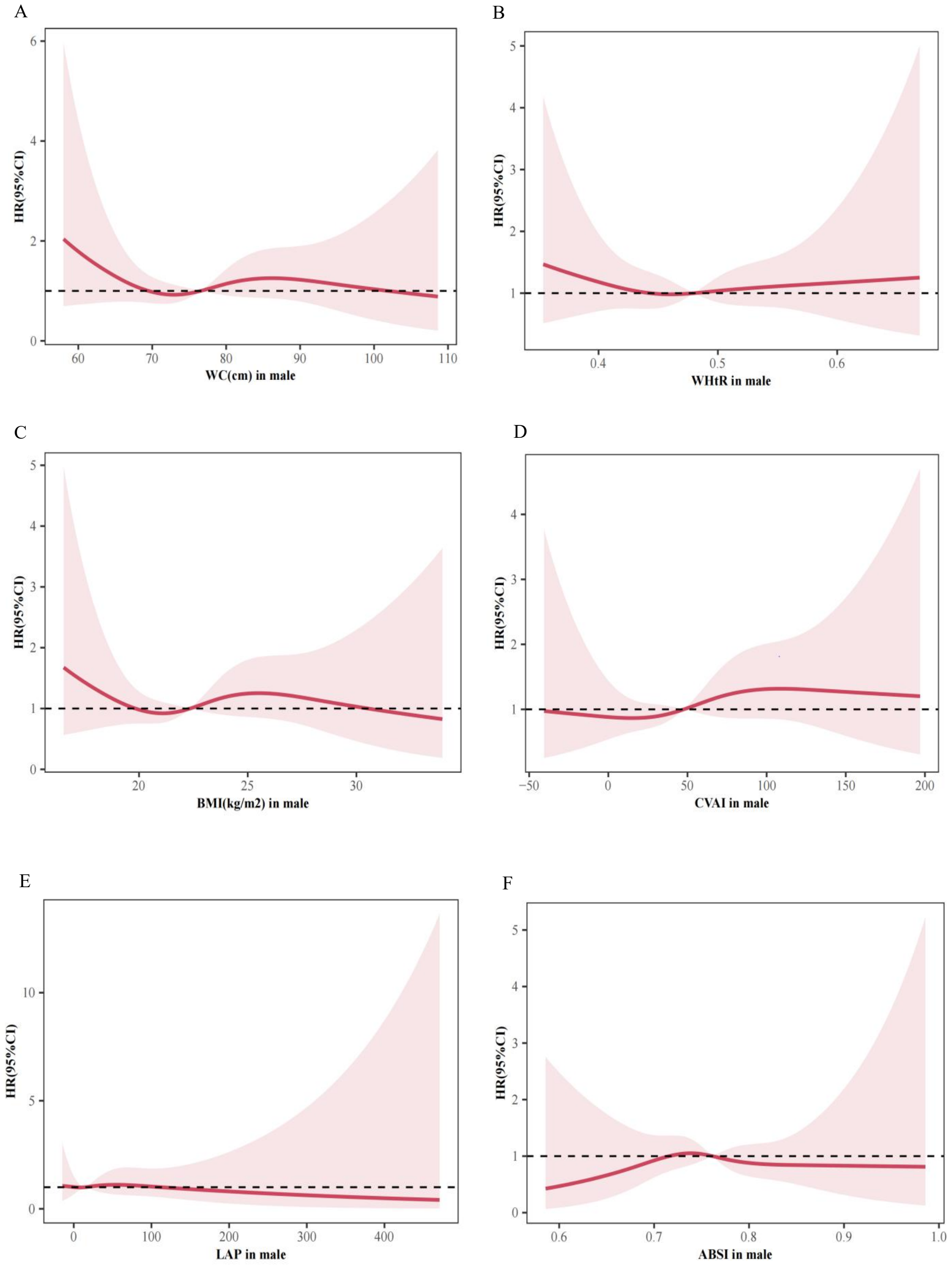

Figure S1 Restricted cubic splines (RCS) for estimating the relationship of overall CVD with traditional and non-traditional anthropometric indicators among male: A. for waist circumference (WC); B. for waist-to-height ratio (WHtR); C. body mass index (BMI); D. Chinese visceral adiposity index (CVAI); E. lipid accumulation product (LAP); F. body shape index (ABSI).

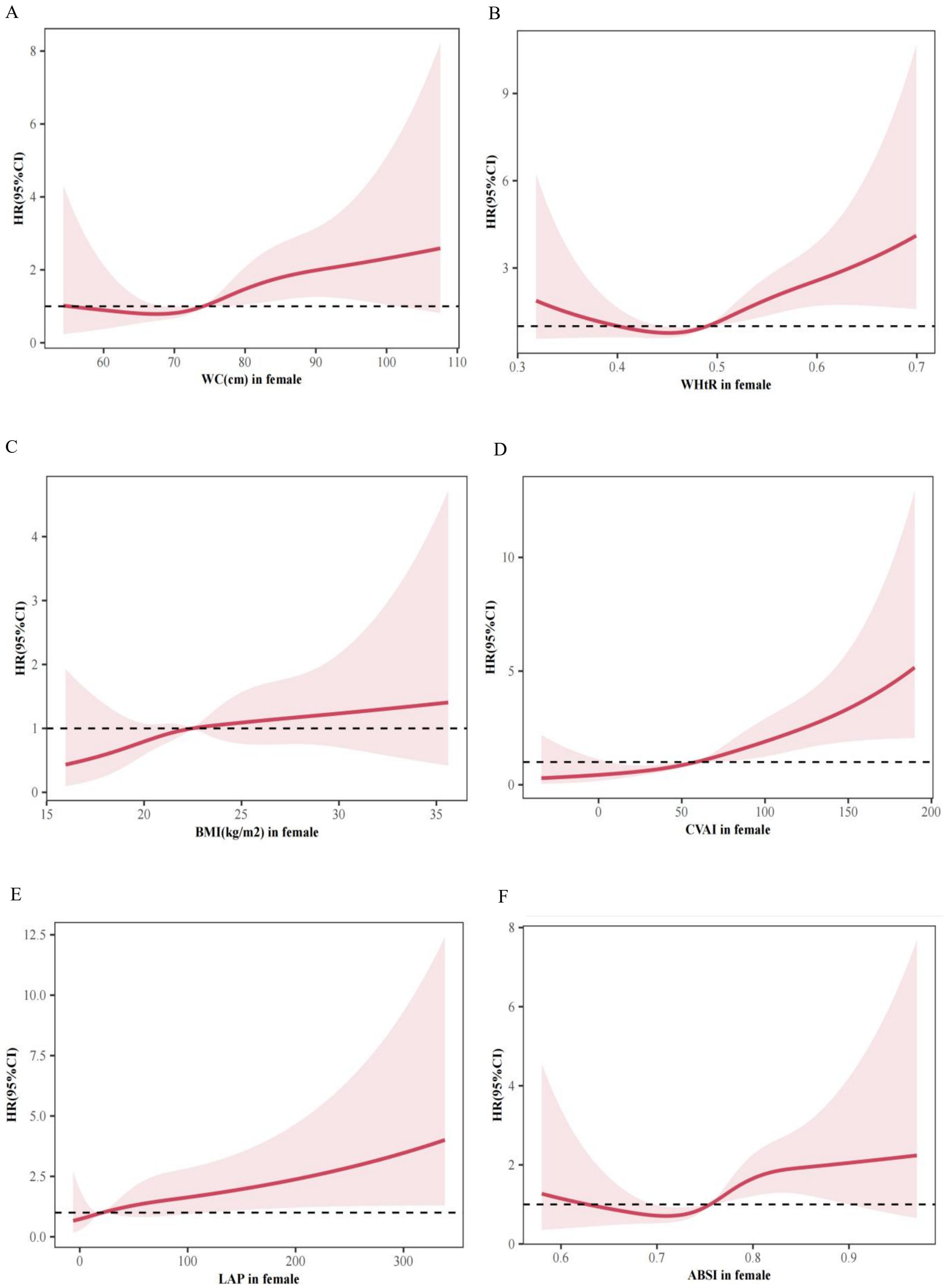

Figure S2 Restricted cubic splines (RCS) for estimating the relationship of overall CVD with traditional and non-traditional anthropometric indicators among female: A. for waist circumference (WC); B. for waist-to-height ratio (WHtR); C. body mass index (BMI); D. Chinese visceral adiposity index (CVAI); E. lipid accumulation product (LAP); F. body shape index (ABSI).

Table S1 Hazard ratios (HRs) and 95% confidence intervals (95%CIs) for ischemic stroke associated with traditional and non-traditional anthropometric indicators among male (n=3717) according to cox regression models

Anthropometric indexes	No (n)	Cases (n)	Incident density (cases per 1000 PYs)	HR(95%CI) [a]		
				Model 1	Model 2	Model 3
**Traditional**						
WC (cm) [b]						
Quartile 1 (Q1)	843	11	1.81	1.00	1.00	1.00
Quartile 2 (Q2)	914	12	1.82	1.00(0.44-2.27)	0.84(0.36-1.94)	0.84(0.36-1.94)
Quartile 3 (Q3)	857	18	2.92	1.64(0.78-3.48)	1.46(0.68-3.13)	1.49(0.69-3.20)
Quartile 4 (Q4)	900	16	2.50	1.40(0.65-3.01)	1.03(0.46-2.31)	1.04(0.46-2.33)
P trend	-	-	-	0.224	0.623	0.606
Per 1 SD	-	-	-	1.11(0.86-1.42)	0.99(0.75-1.30)	0.99(0.75-1.30)
WHtR [c]						
Quartile 1 (Q1)	878	13	2.05	1.00	1.00	1.00
Quartile 2 (Q2)	879	13	2.04	1.01(0.47-2.18)	0.87(0.40-1.91)	0.88(0.40-1.93)
Quartile 3 (Q3)	878	14	2.23	1.12(0.53-2.39)	0.98(0.46-2.11)	0.99(0.46-2.14)
Quartile 4 (Q4)	879	17	2.72	1.37(0.66-2.81)	1.03(0.48-2.21)	1.04(0.48-2.24)
P trend	-	-	-	0.365	0.877	0.855
Per 1 SD	-	-	-	1.08(0.84-1.39)	0.97(0.74-1.27)	0.97(0.74-1.28)
BMI (kg/m^2) [d]						
Quartile 1 (Q1)	929	16	2.41	1.00	1.00	1.00
Quartile 2 (Q2)	929	15	2.25	0.93(0.46-1.88)	0.81(0.39-1.66)	0.80(0.39-1.65)
Quartile 3 (Q3)	925	15	2.28	0.93(0.46-1.89)	0.80(0.39-1.64)	0.80(0.39-1.64)
Quartile 4 (Q4)	934	18	2.72	1.12(0.57-2.20)	0.84(0.41-1.71)	0.84(0.41-1.71)
P trend	-	-	-	0.741	0.653	0.651
Per 1 SD	-	-	-	1.05(0.83-1.34)	0.94(0.72-1.22)	0.94(0.72-1.22)
**Non-traditional**						
CAVI [e]						
Quartile 1 (Q1)	864	9	1.45	1.00	1.00	1.00
Quartile 2 (Q2)	861	13	2.08	1.43(0.61-3.35)	1.25(0.52-2.97)	1.25(0.53-2.99)
Quartile 3 (Q3)	864	13	2.09	1.46(0.62-3.40)	1.26(0.53-3.02)	1.27(0.53-3.04)
Quartile 4 (Q4)	865	21	3.43	2.39(1.09-5.21)	1.82(0.77-4.27)	1.84(0.79-4.32)
P trend	-	-	-	0.028	0.156	0.149
Per 1 SD	-	-	-	1.25(0.98-1.61)	1.10(0.83-1.46)	1.11(0.84-1.47)
LAP [f]						
Quartile 1 (Q1)	869	10	1.60	1.00	1.00	1.00
Quartile 2 (Q2)	863	12	1.94	1.22(0.53-2.83)	1.12(0.48-2.59)	1.11(0.48-2.57)
Quartile 3 (Q3)	868	22	3.53	2.26(1.07-4.77)	1.81(0.84-3.91)	1.79(0.83-3.87)
Quartile 4 (Q4)	872	12	1.91	1.20(0.52-2.78)	0.85(0.34-2.08)	0.85(0.35-2.10)
P trend	-	-	-	0.337	0.156	0.149
Per 1 SD	-	-	-	1.00(0.77-1.30)	0.87(0.63-1.21)	0.88(0.64-1.22)
ABSI [g]						
Quartile 1 (Q1)	878	10	1.58	1.00	1.00	1.00
Quartile 2 (Q2)	876	18	2.87	1.86(0.86-4.02)	1.98(0.89-4.42)	1.98(0.89-4.43)
Quartile 3 (Q3)	876	16	2.52	1.64(0.75-3.62)	1.65(0.72-3.75)	1.67(0.73-3.79)
Quartile 4 (Q4)	876	13	2.08	1.39(0.61-3.17)	1.26(0.53-2.98)	1.28(0.54-3.03)
P trend	-	-	-	0.562	0.827	0.790
Per 1 SD	-	-	-	1.12(0.87-1.44)	1.07(0.82-1.40)	1.08(0.82-1.41)

Abbreviation: WC, waist circumference; WHtR, waist-to-height ratio; BMI, body mass index; CVAI, Chinese visceral adiposity index; LAP, lipid accumulation product; ABSI, body shape index; PYs, person-years; MET, metabolic equivalent of task.

[a] **Model 1:** Adjusted for age only; **Model 2:** Model 1 + additionally adjusted for area, ethnic group. smoking, alcohol drinking, MET, diabetes, hypertension and dyslipidemia; **Model 3**: Model 2 + additionally adjusted for medication use and nutraceutical intake.

[b] **WC (cm):** Quartile levels as Q1, <69.00 cm; Q2, 69.00-74.19 cm; Q3, 74.20-80.99 cm; Q4, ≥81.00 cm;

[c] **WHtR:** Quartile levels as Q1, <0.45; Q2, 0.45-0.48; Q3, 0.49-0.52; Q4, ≥0.53;

[d] **BMI (kg/m^2):** Quartile levels as Q1, <20.59 kg/m^2; Q2, 20.59-22.47 kg/m^2; Q3, 22.48-24.95 kg/m^2; Q4, ≥24.96 kg/m^2;

[e] **CAVI :** Quartile levels as Q1, <30.46; Q2,30.46-58.53; Q3, 58.53-91.25; Q4, ≥91.25;

[f] **LAP:** Quartile levels as Q1, <11.20; Q2, 11.20-20.93; Q3, 20.93-40.00; Q4, ≥40.00;

[g] **ABSI:** Quartile levels as Q1, <0.72; Q2, 0.72- 0.76; Q3, 0.76-0.79; Q4, ≥0.79.

Table S2 Hazard ratios (HRs) and 95% confidence intervals (95%CIs) for ischemic stroke associated with traditional and non-traditional anthropometric indicators among female (n=4120) according to cox regression models

Anthropometric indexes	No (n)	Cases (n)	Incident density (cases per 1000 PYs)	HR(95%CI) [a]		
				Model 1	Model 2	Model 3
**Traditional**						
WC (cm) [b]						
Quartile 1 (Q1)	935	12	1.76	1.00	1.00	1.00
Quartile 2 (Q2)	974	18	2.56	1.46(0.70-3.02)	1.40(0.67-2.90)	1.39(0.67-2.90)
Quartile 3 (Q3)	962	11	1.59	0.92(0.40-2.07)	0.79(0.34-1.80)	0.79(0.34-1.80)
Quartile 4 (Q4)	963	30	4.41	2.55(1.31-4.98)**	1.88(0.93-3.83)	1.88(0.92-3.82)
P trend	-	-	-	0.010	0.142	0.144
Per 1 SD	-	-	-	1.36(1.10-1.69)**	1.22(0.96-1.55)	1.22(0.96-1.55)
WHtR [c]						
Quartile 1 (Q1)	959	12	1.71	1.00	1.00	1.00
Quartile 2 (Q2)	957	15	2.17	1.29(0.60-2.75)	1.24(0.58-2.64)	1.24(0.58-2.66)
Quartile 3 (Q3)	959	14	2.05	1.22(0.56-2.64)	1.09(0.50-2.37)	1.08(0.50-2.36)
Quartile 4 (Q4)	959	30	4.42	2.65(1.36-5.18)**	2.00(0.98-4.07)	2.01(0.99-4.09)
P trend	-	-	-	0.003	0.061	0.06
Per 1 SD	-	-	-	1.48(1.20-1.84)***	1.36(1.07-1.72)*	1.36(1.07-1.72)*
BMI (kg/m^2) [d]						
Quartile 1 (Q1)	1030	18	2.43	1.00	1.00	1.00
Quartile 2 (Q2)	1030	13	1.76	0.73(0.36-1.48)	0.73(0.36-1.49)	0.73(0.36-1.49)
Quartile 3 (Q3)	1030	20	2.75	1.14(0.60-2.15)	1.01(0.53-1.93)	1.02(0.53-1.94)
Quartile 4 (Q4)	1030	26	3.6	1.49(0.82-2.72)	1.09(0.57-2.09)	1.09(0.57-2.08)
P trend	-	-	-	0.092	0.59	0.592
Per 1 SD	-	-	-	1.19(0.97-1.47)	1.07(0.85-1.34)	1.07(0.85-1.34)
**Non-traditional**						
CAVI [e]						
Quartile 1 (Q1)	941	10	1.46	1.00	1.00	1.00
Quartile 2 (Q2)	938	9	1.31	0.90(0.37-2.22)	0.91(0.37-2.24)	0.90(0.37-2.23)
Quartile 3 (Q3)	940	14	2.09	1.43(0.64-3.23)	1.46(0.64-3.33)	1.45(0.63-3.30)
Quartile 4 (Q4)	940	36	5.42	3.72(1.85-7.51)***	3.43(1.57-7.48)**	3.40(1.56-7.44)**
P trend	-	-	-	<0.001	<0.001	<0.001
Per 1 SD	-	-	-	1.83(1.46-2.29)***	1.81(1.39-2.37)***	1.81(1.38-2.36)***
LAP [f]						
Quartile 1 (Q1)	944	13	1.91	1.00	1.00	1.00
Quartile 2 (Q2)	947	15	2.21	1.15(0.55-2.42)	1.11(0.53-2.34)	1.12(0.53-2.35)
Quartile 3 (Q3)	944	16	2.36	1.24(0.60-2.58)	1.09(0.52-2.30)	1.08(0.51-2.28)
Quartile 4 (Q4)	950	25	3.65	1.90(0.97-3.72)	1.61(0.77-3.36)	1.60(0.77-3.35)
P trend	-	-	-	0.051	0.218	0.224
Per 1 SD	-	-	-	1.24(1.10-1.40)***	1.23(1.07-1.41)**	1.24(1.07-1.43)**
ABSI [g]						
Quartile 1 (Q1)	959	12	1.73	1.00	1.00	1.00
Quartile 2 (Q2)	956	16	2.31	1.34(0.64-2.84)	1.33(0.63-2.82)	1.34(0.63-2.84)
Quartile 3 (Q3)	957	15	2.19	1.29(0.60-2.75)	1.18(0.55-2.54)	1.20(0.56-2.58)
Quartile 4 (Q4)	958	28	4.10	2.43(1.23-4.77)*	1.94(0.98-3.87)*	1.96(0.99-3.90)*
P trend	-	-	-	0.010	0.067	0.063
Per 1 SD	-	-	-	1.37(1.10-1.70)**	1.28(1.02-1.61)*	1.28(1.02-1.61)*

Abbreviation: WC, waist circumference; WHtR, waist-to-height ratio; BMI, body mass index; CVAI, Chinese visceral adiposity index; LAP, lipid accumulation product; ABSI, body shape index; PYs, person-years; MET, metabolic equivalent of task.

[a] **Model 1:** Adjusted for age only; **Model 2:** Model 1 + additionally adjusted for area, ethnic group. smoking, alcohol drinking, MET, diabetes, hypertension and dyslipidemia; **Model 3**: Model 2 + additionally adjusted for medication use and nutraceutical intake.

[b] **WC (cm):** Quartile levels as Q1, <69.00 cm; Q2, 69.00-74.19 cm; Q3, 74.20-80.99 cm; Q4, ≥81.00 cm;

[c] **WHtR:** Quartile levels as Q1, <0.45; Q2, 0.45-0.48; Q3, 0.49-0.52; Q4, ≥0.53;

[d] **BMI (kg/m^2):** Quartile levels as Q1, <20.59 kg/m^2; Q2, 20.59-22.47 kg/m^2; Q3, 22.48-24.95 kg/m^2; Q4, ≥24.96 kg/m^2;

[e] **CAVI :** Quartile levels as Q1, <30.46; Q2,30.46-58.53; Q3, 58.53-91.25; Q4, ≥91.25;

[f] **LAP:** Quartile levels as Q1, <11.20; Q2, 11.20-20.93; Q3, 20.93-40.00; Q4, ≥40.00;

[g] **ABSI:** Quartile levels as Q1, <0.72; Q2, 0.72- 0.76; Q3, 0.76-0.79; Q4, ≥0.79.

* *P*<0.05; ** 0.05<*P*<0.01; *** *P*<0.001.

Table S3 Hazard ratios (HRs) and 95% confidence intervals (95%CIs) for haemorrhagic stroke associated with traditional and non-traditional anthropometric indicators among male (n=3717) according to cox regression models

Anthropometric indexes	No (n)	Cases (n)	Incident density (cases per 1000 PYs)	HR(95%CI) [a]		
				Model 1	Model 2	Model 3
**Traditional**						
WC (cm) [b]						
Quartile 1 (Q1)	843	7	1.15	1.00	1.00	1.00
Quartile 2 (Q2)	914	8	1.21	1.05(0.38-2.91)	0.98(0.35-2.71)	0.98(0.35-2.72)
Quartile 3 (Q3)	857	4	0.64	0.57(0.17-1.96)	0.50(0.14-1.74)	0.49(0.14-1.71)
Quartile 4 (Q4)	900	5	0.78	0.69(0.22-2.17)	0.54(0.16-1.83)	0.54(0.16-1.83)
P trend	-	-	-	0.354	0.206	0.201
Per 1 SD	-	-	-	0.79(0.51-1.21)	0.71(0.45-1.12)	0.71(0.45-1.12)
WHtR [c]						
Quartile 1 (Q1)	878	9	1.42	1.00	1.00	1.00
Quartile 2 (Q2)	879	7	1.10	0.79(0.29-2.12)	0.71(0.26-1.92)	0.70(0.26-1.90)
Quartile 3 (Q3)	878	4	0.64	0.47(0.14-1.52)	0.39(0.12-1.28)	0.38(0.12-1.26)
Quartile 4 (Q4)	879	4	0.64	0.47(0.14-1.52)	0.33(0.10-1.16)	0.33(0.09-1.15)
P trend	-	-	-	0.128	0.047	0.045
Per 1 SD	-	-	-	0.78(0.51-1.19)	0.69(0.44-1.07)	0.69(0.44-1.07)
BMI (kg/m^2) [d]						
Quartile 1 (Q1)	929	5	0.75	1.00	1.00	1.00
Quartile 2 (Q2)	929	6	0.90	1.19(0.36-3.89)	1.12(0.34-3.69)	1.13(0.34-3.71)
Quartile 3 (Q3)	925	9	1.37	1.80(0.60-5.37)	1.58(0.52-4.80)	1.57(0.52-4.76)
Quartile 4 (Q4)	934	5	0.75	1.00(0.29-3.46)	0.79(0.22-2.87)	0.80(0.22-2.90)
P trend	-	-	-	0.781	0.897	0.901
Per 1 SD	-	-	-	0.95(0.64-1.43)	0.86(0.56-1.32)	0.86(0.56-1.33)
**Non-traditional**						
CAVI [e]						
Quartile 1 (Q1)	865	4	0.64	1.00	1.00	1.00
Quartile 2 (Q2)	865	5	0.80	1.23(0.33-4.57)	1.22(0.33-4.58)	1.22(0.33-4.58)
Quartile 3 (Q3)	865	11	1.76	2.77(0.88-8.71)	2.53(0.78-8.26)	2.52(0.77-8.21)
Quartile 4 (Q4)	866	4	0.65	1.01(0.25-4.04)	0.86(0.20-3.76)	0.85(0.20-3.75)
P trend	-	-	-	0.561	0.776	0.783
Per 1 SD	-	-	-	0.96(0.64-1.44)	0.87(0.55-1.36)	0.86(0.55-1.36)
LAP [f]						
Quartile 1 (Q1)	870	5	0.80	1.00	1.00	1.00
Quartile 2 (Q2)	868	8	1.29	1.61(0.53-4.92)	1.49(0.48-4.58)	1.50(0.49-4.62)
Quartile 3 (Q3)	869	9	1.44	1.84(0.62-5.5)	1.58(0.51-4.84)	1.60(0.52-4.93)
Quartile 4 (Q4)	872	2	0.32	0.4(0.08-2.06)	0.29(0.05-1.59)	0.29(0.05-1.59)
P trend	-	-	-	0.475	0.279	0.278
Per 1 SD	-	-	-	0.61(0.27-1.38)	0.49(0.20-1.21)	0.48(0.20-1.20)
ABSI [g]						
Quartile 1 (Q1)	879	9	1.42	1.00	1.00	1.00
Quartile 2 (Q2)	878	7	1.11	0.80(0.30-2.15)	0.76(0.28-2.05)	0.77(0.28-2.07)
Quartile 3 (Q3)	878	3	0.47	0.34(0.09-1.27)	0.31(0.08-1.17)	0.31(0.08-1.16)
Quartile 4 (Q4)	879	5	0.80	0.60(0.20-1.78)	0.52(0.17-1.58)	0.51(0.17-1.56)
P trend	-	-	-	0.183	0.120	0.115
Per 1 SD	-	-	-	0.77(0.51-1.15)	0.74(0.49-1.11)	0.74(0.49-1.11)

Abbreviation: WC, waist circumference; WHtR, waist-to-height ratio; BMI, body mass index; CVAI, Chinese visceral adiposity index; LAP, lipid accumulation product; ABSI, body shape index; PYs, person-years; MET, metabolic equivalent of task.

[a] **Model 1:** Adjusted for age only; **Model 2:** Model 1 + additionally adjusted for area, ethnic group. smoking, alcohol drinking, MET, diabetes, hypertension and dyslipidemia; **Model 3**: Model 2 + additionally adjusted for medication use and nutraceutical intake.

[b] **WC (cm):** Quartile levels as Q1, <69.00 cm; Q2, 69.00-74.19 cm; Q3, 74.20-80.99 cm; Q4, ≥81.00 cm;

[c] **WHtR:** Quartile levels as Q1, <0.45; Q2, 0.45-0.48; Q3, 0.49-0.52; Q4, ≥0.53;

[d] **BMI (kg/m^2):** Quartile levels as Q1, <20.59 kg/m^2; Q2, 20.59-22.47 kg/m^2; Q3, 22.48-24.95 kg/m^2; Q4, ≥24.96 kg/m^2;

[e] **CAVI :** Quartile levels as Q1, <30.46; Q2,30.46-58.53; Q3, 58.53-91.25; Q4, ≥91.25;

[f] **LAP:** Quartile levels as Q1, <11.20; Q2, 11.20-20.93; Q3, 20.93-40.00; Q4, ≥40.00;

[g] **ABSI:** Quartile levels as Q1, <0.72; Q2, 0.72- 0.76; Q3, 0.76-0.79; Q4, ≥0.79.

Table S4 Hazard ratios (HRs) and 95% confidence intervals (95%CIs) for haemorrhagic stroke associated with traditional and non-traditional anthropometric indicators among female (n=4120) according to cox regression models

Anthropometric indexes	No (n)	Cases (n)	Incident density (cases per 1000 PYs)	HR(95%CI) [a]		
				Model 1	Model 2	Model 3
**Traditional**						
WC (cm) [b]						
Quartile 1 (Q1)	935	2	0.29	1.00	1.00	1.00
Quartile 2 (Q2)	974	3	0.42	1.46(0.24-8.72)	1.35(0.23-8.11)	1.35(0.23-8.12)
Quartile 3 (Q3)	962	9	1.31	4.48(0.97-20.80)	3.69(0.78-17.50)	3.79(0.80-17.90)
Quartile 4 (Q4)	963	6	0.88	2.99(0.60-14.80)	2.14(0.41-11.30)	2.11(0.40-11.10)
P trend	-	-	-	0.075	0.244	0.249
Per 1 SD	-	-	-	1.58(1.07-2.32)*	1.42(0.93-2.17)	1.42(0.93-2.18)
WHtR [c]						
Quartile 1 (Q1)	959	1	0.14	1.00	1.00	1.00
Quartile 2 (Q2)	957	3	0.43	3.10(0.32-29.80)	2.85(0.30-27.60)	2.88(0.30-27.80)
Quartile 3 (Q3)	959	8	1.17	8.33(1.04-66.60)*	7.54(0.93-60.80)	7.62(0.94-61.60)
Quartile 4 (Q4)	959	8	1.18	8.29(1.04-66.30)*	5.96(0.71-50.00)	5.95(0.71-50.00)
P trend	-	-	-	0.011	0.050	0.051
Per 1 SD	-	-	-	1.72(1.18-2.52)**	1.53(1.00-2.33)	1.54(1.00-2.35)
BMI (kg/m^2) [d]						
Quartile 1 (Q1)	1030	5	0.67	1.00	1.00	1.00
Quartile 2 (Q2)	1030	5	0.68	1.01(0.29-3.47)	0.97(0.28-3.37)	0.98(0.28-3.39)
Quartile 3 (Q3)	1030	5	0.68	1.01(0.29-3.47)	0.84(0.24-2.96)	0.84(0.24-2.96)
Quartile 4 (Q4)	1030	6	0.83	1.21(0.37-3.95)	0.82(0.23-2.92)	0.82(0.23-2.93)
P trend	-	-	-	0.764	0.722	0.721
Per 1 SD	-	-	-	1.12(0.75-1.68)	0.97(0.63-1.51)	0.97(0.63-1.51)
**Non-traditional**						
CAVI [e]						
Quartile 1 (Q1)	941	1	0.15	1.00	1.00	1.00
Quartile 2 (Q2)	939	1	0.15	0.99(0.06-15.80)	0.95(0.06-15.20)	0.94(0.06-15.10)
Quartile 3 (Q3)	941	7	1.04	6.98(0.86-56.70)	7.14(0.86-59.60)	7.09(0.85-59.30)
Quartile 4 (Q4)	941	11	1.64	10.9(1.41-84.40)*	11.00(1.28-95.20)*	10.8(1.25-93.4)*
P trend	-	-	-	0.001	0.003	0.003
Per 1 SD	-	-	-	1.99(1.32-3.01)**	1.96(1.19-3.23)*	1.94(1.18-3.20)*
LAP [f]						
Quartile 1 (Q1)	945	1	0.15	1.00	1.00	1.00
Quartile 2 (Q2)	947	5	0.74	4.98(0.58-42.70)	4.98(0.58-42.80)	4.95(0.58-42.50)
Quartile 3 (Q3)	944	5	0.73	4.99(0.58-42.70)	4.58(0.52-40.20)	4.53(0.52-39.80)
Quartile 4 (Q4)	952	9	1.31	8.67(1.10-68.50)*	7.92(0.92-68.20)	7.85(0.91-67.60)
P trend	-	-	-	0.024	0.058	0.059
Per 1 SD	-	-	-	1.13(0.83-1.53)	1.05(0.72-1.55)	1.04(0.71-1.53)
ABSI [g]						
Quartile 1 (Q1)	959	1	0.14	1.00	1.00	1.00
Quartile 2 (Q2)	957	4	0.57	4.03(0.45-36.00)	3.77(0.42-33.80)	3.76(0.42-33.80)
Quartile 3 (Q3)	958	4	0.58	4.13(0.46-37.00)	3.64(0.40-32.80)	3.67(0.41-33.10)
Quartile 4 (Q4)	959	11	1.60	11.6(1.49-89.60)*	9.31(1.19-72.90)*	9.29(1.19-72.80)*
P trend	-	-	-	0.004	0.010	0.011
Per 1 SD	-	-	-	1.71(1.19-2.46)**	1.68(1.13-2.51)*	1.67(1.12-2.50)*

Abbreviation: WC, waist circumference; WHtR, waist-to-height ratio; BMI, body mass index; CVAI, Chinese visceral adiposity index; LAP, lipid accumulation product; ABSI, body shape index; PYs, person-years; MET, metabolic equivalent of task.

[a] **Model 1:** Adjusted for age only; **Model 2:** Model 1 + additionally adjusted for area, ethnic group. smoking, alcohol drinking, MET, diabetes, hypertension and dyslipidemia; **Model 3**: Model 2 + additionally adjusted for medication use and nutraceutical intake.

[b] **WC (cm):** Quartile levels as Q1, <69.00 cm; Q2, 69.00-74.19 cm; Q3, 74.20-80.99 cm; Q4, ≥81.00 cm;

[c] **WHtR:** Quartile levels as Q1, <0.45; Q2, 0.45-0.48; Q3, 0.49-0.52; Q4, ≥0.53;

[d] **BMI (kg/m^2):** Quartile levels as Q1, <20.59 kg/m^2; Q2, 20.59-22.47 kg/m^2; Q3, 22.48-24.95 kg/m^2; Q4, ≥24.96 kg/m^2;

[e] **CAVI :** Quartile levels as Q1, <30.46; Q2,30.46-58.53; Q3, 58.53-91.25; Q4, ≥91.25;

[f] **LAP:** Quartile levels as Q1, <11.20; Q2, 11.20-20.93; Q3, 20.93-40.00; Q4, ≥40.00;

[g] **ABSI:** Quartile levels as Q1, <0.72; Q2, 0.72- 0.76; Q3, 0.76-0.79; Q4, ≥0.79.

* *P*<0.05; ** 0.05<*P*<0.01.

Table S5 Hazard ratios (HRs) and 95% confidence intervals (95%CIs) for myocardial infarction associated with traditional and non-traditional anthropometric indicators among male (n=3717) according to cox regression models

Anthropometric indexes	No (n)	Cases (n)	Incident density (cases per 1000 PYs)	HR(95%CI) [a]		
				Model 1	Model 2	Model 3
**Traditional**						
WC (cm) [b]						
Quartile 1 (Q1)	843	6	0.99	1.00	1.00	1.00
Quartile 2 (Q2)	914	1	0.15	0.15(0.02-1.26)	0.17(0.02-1.46)	0.16(0.02-1.41)
Quartile 3 (Q3)	857	2	0.32	0.34(0.07-1.70)	0.36(0.07-1.89)	0.35(0.06-1.83)
Quartile 4 (Q4)	900	4	0.62	0.65(0.18-2.31)	0.66(0.16-2.74)	0.65(0.15-2.70)
P trend	-	-	-	0.55	0.641	0.630
Per 1 SD	-	-	-	0.89(0.50-1.57)	0.96(0.53-1.75)	0.96(0.52-1.75)
WHtR [c]						
Quartile 1 (Q1)	878	5	0.79	1.00	1.00	1.00
Quartile 2 (Q2)	879	2	0.31	0.42(0.08-2.15)	0.48(0.09-2.63)	0.46(0.08-2.55)
Quartile 3 (Q3)	878	1	0.16	0.22(0.03-1.92)	0.25(0.03-2.23)	0.24(0.03-2.17)
Quartile 4 (Q4)	879	5	0.80	1.12(0.32-3.89)	1.19(0.29-4.93)	1.18(0.28-4.90)
P trend	-	-	-	0.962	0.879	0.885
Per 1 SD	-	-	-	0.98(0.57-1.71)	1.04(0.58-1.86)	1.03(0.57-1.86)
BMI (kg/m^2) [d]						
Quartile 1 (Q1)	929	5	0.75	1.00	1.00	1.00
Quartile 2 (Q2)	929	3	0.45	0.58(0.14-2.43)	0.72(0.16-3.25)	0.73(0.16-3.30)
Quartile 3 (Q3)	925	4	0.61	0.79(0.21-2.95)	0.89(0.22-3.63)	0.88(0.21-3.59)
Quartile 4 (Q4)	934	2	0.30	0.40(0.08-2.06)	0.39(0.07-2.30)	0.40(0.07-2.31)
P trend	-	-	-	0.346	0.376	0.376
Per 1 SD	-	-	-	0.92(0.53-1.61)	0.97(0.55-1.72)	0.98(0.55-1.73)
**Non-traditional**						
CAVI [e]						
Quartile 1 (Q1)	865	17	2.74	1.00	1.00	1.00
Quartile 2 (Q2)	865	18	2.89	0.38(0.07-1.96)	0.49(0.09-2.68)	0.48(0.09-2.63)
Quartile 3 (Q3)	865	23	3.70	0.20(0.02-1.68)	0.22(0.02-2.04)	0.22(0.02-2.01)
Quartile 4 (Q4)	866	28	4.59	1.01(0.29-3.48)	1.07(0.24-4.74)	1.06(0.24-4.72)
P trend	-	-	-	0.346	0.991	0.982
Per 1 SD	-	-	-	1.13(0.66-1.93)	1.17(0.64-2.13)	1.17(0.64-2.13)
LAP [f]						
Quartile 1 (Q1)	870	20	3.20	1.00	1.00	1.00
Quartile 2 (Q2)	868	18	2.91	0.17(0.02-1.39)	0.19(0.02-1.66)	0.19(0.02-1.62)
Quartile 3 (Q3)	869	30	4.83	0.17(0.02-1.45)	0.20(0.02-1.72)	0.19(0.02-1.66)
Quartile 4 (Q4)	872	18	2.88	0.83(0.25-2.72)	0.87(0.21-3.61)	0.85(0.20-3.55)
P trend	-	-	-	0.72	0.809	0.787
Per 1 SD	-	-	-	1.09(0.75-1.57)	1.07(0.74-1.54)	1.07(0.75-1.54)
ABSI [g]						
Quartile 1 (Q1)	879	21	3.33	1.00	1.00	1.00
Quartile 2 (Q2)	878	25	4.00	1.08(0.22-5.34)	1.43(0.24-8.66)	1.42(0.24-8.60)
Quartile 3 (Q3)	878	22	3.47	1.80(0.43-7.53)	2.36(0.45-12.40)	2.34(0.45-12.30)
Quartile 4 (Q4)	879	19	3.04	0.79(0.13-4.73)	1.03(0.14-7.59)	1.02(0.14-7.53)
P trend	-	-	-	0.72	0.774	0.781
Per 1 SD	-	-	-	0.89(0.51-1.55)	0.91(0.49-1.68)	0.90(0.49-1.68)

Abbreviation: WC, waist circumference; WHtR, waist-to-height ratio; BMI, body mass index; CVAI, Chinese visceral adiposity index; LAP, lipid accumulation product; ABSI, body shape index; PYs, person-years; MET, metabolic equivalent of task.

[a] **Model 1:** Adjusted for age only; **Model 2:** Model 1 + additionally adjusted for area, ethnic group, smoking, alcohol drinking, MET, diabetes, hypertension and dyslipidemia; **Model 3**: Model 2 + additionally adjusted for medication use and nutraceutical intake.

[b] **WC (cm):** Quartile levels as Q1, <69.00 cm; Q2, 69.00-74.19 cm; Q3, 74.20-80.99 cm; Q4, ≥81.00 cm;

[c] **WHtR:** Quartile levels as Q1, <0.45; Q2, 0.45-0.48; Q3, 0.49-0.52; Q4, ≥0.53;

[d] **BMI (kg/m^2):** Quartile levels as Q1, <20.59 kg/m^2; Q2, 20.59-22.47 kg/m^2; Q3, 22.48-24.95 kg/m^2; Q4, ≥24.96 kg/m^2;

[e] **CAVI :** Quartile levels as Q1, <30.46; Q2,30.46-58.53; Q3, 58.53-91.25; Q4, ≥91.25;

[f] **LAP:** Quartile levels as Q1, <11.20; Q2, 11.20-20.93; Q3, 20.93-40.00; Q4, ≥40.00;

[g] **ABSI:** Quartile levels as Q1, <0.72; Q2, 0.72- 0.76; Q3, 0.76-0.79; Q4, ≥0.79.

Table S6 Hazard ratios (HRs) and 95% confidence intervals (95%CIs) for myocardial infarction associated with traditional and non-traditional anthropometric indicators among female (n=4120) according to cox regression models

Anthropometric indexes	No (n)	Cases (n)	Incident density (cases per 1000 PYs)	HR(95%CI) [a]		
				Model 1	Model 2	Model 3
**Traditional**						
WC (cm) [b]						
Quartile 1 (Q1)	935	3	0.44	1.00	1.00	1.00
Quartile 2 (Q2)	974	1	0.14	0.34(0.04-3.28)	0.29(0.03-2.84)	0.29(0.03-2.84)
Quartile 3 (Q3)	962	3	0.43	1.07(0.22-5.31)	0.81(0.16-4.18)	0.80(0.16-4.16)
Quartile 4 (Q4)	963	4	0.58	1.46(0.33-6.52)	0.84(0.17-4.32)	0.85(0.17-4.33)
P trend	-	-	-	0.432	0.886	0.887
Per 1 SD	-	-	-	1.07(0.59-1.92)	0.84(0.45-1.58)	0.84(0.45-1.58)
WHtR [c]						
Quartile 1 (Q1)	959	3	0.43	1.00	1.00	1.00
Quartile 2 (Q2)	957	3	0.43	1.12(0.23-5.56)	0.98(0.20-4.87)	0.98(0.20-4.89)
Quartile 3 (Q3)	959	1	0.15	0.38(0.04-3.63)	0.31(0.03-3.05)	0.31(0.03-3.08)
Quartile 4 (Q4)	959	4	0.59	1.53(0.34-6.86)	0.82(0.16-4.29)	0.81(0.16-4.28)
P trend	-	-	-	0.768	0.493	0.495
Per 1 SD	-	-	-	0.92(0.50-1.68)	0.70(0.38-1.32)	0.70(0.38-1.31)
BMI (kg/m^2) [d]						
Quartile 1 (Q1)	1030	5	0.67	1.00	1.00	1.00
Quartile 2 (Q2)	1030	1	0.13	0.20(0.02-1.73)	0.20(0.02-1.72)	0.02(0.02-1.71)
Quartile 3 (Q3)	1030	2	0.27	0.42(0.08-2.15)	0.37(0.07-1.91)	0.36(0.07-1.91)
Quartile 4 (Q4)	1030	5	0.69	1.04(0.30-3.60)	0.67(0.17-2.59)	0.66(0.17-2.57)
P trend	-	-	-	0.851	0.462	0.467
Per 1 SD	-	-	-	1.23(0.75-2.01)	1.04(0.60-1.77)	1.03(0.60-1.77)
**Non-traditional**						
CAVI [e]						
Quartile 1 (Q1)				1.00	1.00	1.00
Quartile 2 (Q2)	937	1	0.15	2.00(0.18-22.10)	1.69(0.15-19.00)	1.70(0.15-19.10)
Quartile 3 (Q3)	929	2	0.29	3.15(0.33-30.30)	2.24(0.22-23.20)	2.25(0.22-23.30)
Quartile 4 (Q4)	928	3	0.44	4.15(0.46-37.20)	1.83(0.16-21.20)	1.83(0.16-21.20)
P trend	928	4	0.60	0.152	0.823	0.817
Per 1 SD	-	-	-	1.60(0.89-2.87)	1.18(0.58-2.42)	1.18(0.58-2.42)
LAP [f]	-	-	-			
Quartile 1 (Q1)				1.00	1.00	1.00
Quartile 2 (Q2)	941	3	0.44	0.68(0.11-4.08)	0.56(0.09-3.42)	0.56(0.09-3.40)
Quartile 3 (Q3)	935	2	0.29	0.34(0.04-3.27)	0.23(0.02-2.32)	0.23(0.02-2.33)
Quartile 4 (Q4)	937	1	0.15	1.29(0.29-5.79)	0.60(0.11-3.29)	0.59(0.11-3.26)
P trend	935	4	0.58	0.807	0.317	0.313
Per 1 SD	-	-	-	0.80(0.31-2.03)	0.49(0.16-1.54)	0.49(0.15-1.53)
ABSI [g]	-	-	-			
Quartile 1 (Q1)				1.00	1.00	1.00
Quartile 2 (Q2)	953	4	0.58	0.76(0.17-3.40)	0.67(0.15-3.01)	0.67(0.15-3.02)
Quartile 3 (Q3)	946	3	0.43	0.28(0.03-2.48)	0.24(0.03-2.18)	0.24(0.03-2.15)
Quartile 4 (Q4)	945	1	0.14	0.85(0.19-3.81)	0.59(0.13-2.74)	0.59(0.13-2.73)
P trend	949	3	0.44	0.616	0.401	0.393
Per 1 SD	-	-	-	0.71(0.40-1.25)	0.65(0.38-1.11)	0.65(0.38-1.1)

Abbreviation: WC, waist circumference; WHtR, waist-to-height ratio; BMI, body mass index; CVAI, Chinese visceral adiposity index; LAP, lipid accumulation product; ABSI, body shape index; PYs, person-years; MET, metabolic equivalent of task.

[a] **Model 1:** Adjusted for age only; **Model 2:** Model 1 + additionally adjusted for area, ethnic group. smoking, alcohol drinking, MET, diabetes, hypertension and dyslipidemia; **Model 3**: Model 2 + additionally adjusted for medication use and nutraceutical intake.

[b] **WC (cm):** Quartile levels as Q1, <69.00 cm; Q2, 69.00-74.19 cm; Q3, 74.20-80.99 cm; Q4, ≥81.00 cm;

[c] **WHtR:** Quartile levels as Q1, <0.45; Q2, 0.45-0.48; Q3, 0.49-0.52; Q4, ≥0.53;

[d] **BMI (kg/m^2):** Quartile levels as Q1, <20.59 kg/m^2; Q2, 20.59-22.47 kg/m^2; Q3, 22.48-24.95 kg/m^2; Q4, ≥24.96 kg/m^2;

[e] **CAVI :** Quartile levels as Q1, <30.46; Q2,30.46-58.53; Q3, 58.53-91.25; Q4, ≥91.25;

[f] **LAP:** Quartile levels as Q1, <11.20; Q2, 11.20-20.93; Q3, 20.93-40.00; Q4, ≥40.00;

[g] **ABSI:** Quartile levels as Q1, <0.72; Q2, 0.72- 0.76; Q3, 0.76-0.79; Q4, ≥0.79.

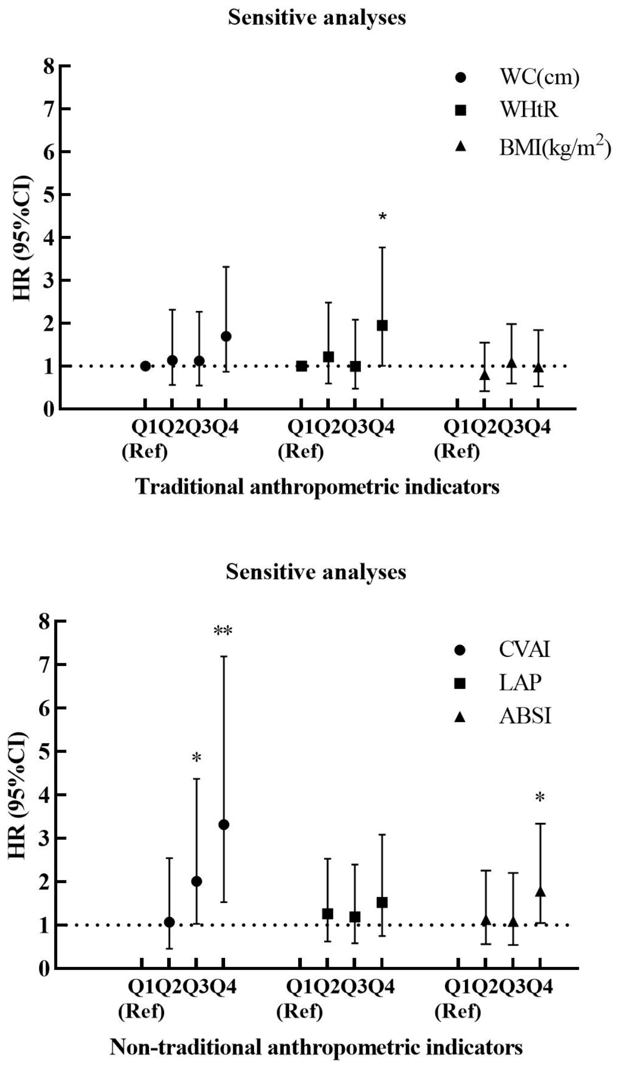

Figure S3 Sensitive analyses based on adjusted cox regression model (Model 3) after excluding female with less than one year of follow-up :A. for waist circumference (WC), waist-to-height ratio (WHtR) and body mass index (BMI), respectively; B. for Chinese visceral adiposity index (CVAI), lipid accumulation product (LAP) and body shape index (ABSI). * $P<0.05$; ** $0.05<P<0.01$.
